# Mechanistic Studies on the Dibenzofuran Formation from Phenanthrene, Fluorene and 9–Fluorenone

**DOI:** 10.3390/ijms16035271

**Published:** 2015-03-06

**Authors:** Shanqing Li, Qingzhu Zhang

**Affiliations:** Environment Research Institute, Shandong University, Jinan 250100, China; E-Mail: lishanqing@mail.sdu.edu.cn

**Keywords:** dibenzofuran, polycyclic aromatic hydrocarbons, formation mechanism, chlorine monoxide radical, density functional method

## Abstract

We carried out molecular orbital theory calculations for the homogeneous gas‑phase formation of dibenzofuran from phenanthrene, fluorene, 9-methylfluorene and 9-fluorenone. Dibenzofuran will be formed if ∙OH adds to C_8a_, and the order of reactivity follows as 9-fluorenone > 9-methylfluorene > fluorene > phenanthrene. The oxidations initiated by ClO∙ are more favorable processes, considering that the standard reaction Gibbs energies are at least 21.63 kcal/mol lower than those of the equivalent reactions initiated by ∙OH. The adding of ∙OH and then O_2_ to phenanthrene is a more favorable route than adding ∙OH to C_8a_ of phenanthrene, when considering the greater reaction extent. The reaction channel from fluorene and O_2_ to 9-fluorenone and H_2_O seems very important, not only because it contains only three elementary reactions, but because the standard reaction Gibbs energies are lower than −80.07 kcal/mol.

## 1. Introduction

Incineration is an efficient means of waste disposal, owing to significant volume reduction and energy recovery [1]. Unfortunately, a large quantity of toxic byproducts, such as polychlorinated dibenzo‑*p*‑dioxins (PCDDs) and polychlorinated dibenzofurans (PCDFs), are emitted from the incinerators [2]. PCDD/Fs are among the most toxic environmental contaminants due to their potency as aryl hydrocarbon receptor (AHR) ligands [3,4]. PCDD/F emissions have caused serious public concern and sparked the controversy about the acceptance of incineration as a viable solid waste treatment method. Suppressing the PCDD/F formations may be more efficient than end-of-pipe technology for PCDD/Fs in incineration in the future. Despite many studies, there remains much uncertainty about the predominant formation pathways of PCDD/Fs in combustion systems.

PCDD/Fs are formed mainly through three pathways in combustion [5,6,7,8,9]: (I) gas-phase formations from molecular precursors at high temperatures (>600 °C); (II) catalytic reactions on the surface of fly ash across temperature range of 200–600 °C; and (III) chlorination and dechlorination of the existing PCDD/Fs. Chlorophenols are generally considered as the predominant precursors of PCDD/Fs and implicated as key intermediates in the *de novo* synthesis [10,11]. However, diversify sources of PCDD/Fs have been demonstrated, such as catechol [12], semiquinone radicals [13] and captan pesticide [14] and polycyclic aromatic hydrocarbons (PAHs) [15]. Both catalytic and non‑catalytic condensations of chlorophenols showed that the ratio of PCDDs and PCDFs either in gas phase or on fly ash is more than one, but PCDFs are major congeners in waste incinerators [11,16,17,18]. The catalytic reaction of amorphous ^12^C- and ^13^C-labeled carbon revealed that approximately half of PCDDs are formed via the condensation of monocyclic aromatic precursors; however, PCDFs are primarily released from the oxidation of the precursors with two or more aromatic rings, such as PAHs and elemental carbon [19]. As evidence, the multiple regression analysis showed that the coefficient of determination between the toxic equivalent (TEQ) of PCDD/Fs and the concentration of naphthalene, fluorene and phenanthrene was 0.85 [20]. PAHs are recognized as potential carbon sources of PCDFs, because the causes are not only the structural similarities, but also high concentrations in fly ash and tail gas. Total amounts of PAHs in the different ash samples vary from 14 to 77,000 μg/kg [21], and the concentrations in gas phase range from 566 to 8374 μg/Nm^3^ [22,23].

The chlorination of dibenzofuran (DF) could play an important role in forming PCDFs. On the one hand, the complete distributions for PCDF congeners shows broadly consistent between the chlorination model and the data from incinerators [6,24]; In addition, the chlorination of DF vapor by copper (II) chloride is proved to be a highly efficient process [24]. On the other, the high concentration of DF supports the hypothesis. The concentration of DF in flue gas is 50–700 times that of total PCDFs, and 2600 times during combustion disturbance [16,25].

Cosentino *et al.* [15] reported that oxidative pathways of pyrene and benzodibenzofuran—a model for the de novo synthesis—are possible routes of dibenzofuran formation. Meanwhile, theoretical studies on the oxidation mechanisms in combustion processes of aromatic and unsaturated hydrocarbons [26,27] suggested that the oxidation of PAHs is a source of DF and dibenzo‑*p*‑dioxin (DD). The nearly identical congener’s distributions of PCDD/Fs among different emission sources serves as an evidence for the occurrence of heterogeneous mechanism similar to the formation mechanism from PAHs. For the further investigation of the DD/DF formation mechanism from PAHs and seeking after the heterogeneous mechanism of PCDD/Fs in incineration processes, we carried out a density functional theory study on the mechanistic aspects of DF formation from phenanthrene (Phe), fluorene (Flu), 9-menthylfluorene (9MF), as well as 9-fluorenone (9FO).

## 2. Results and Discussion

**Scheme 1:**
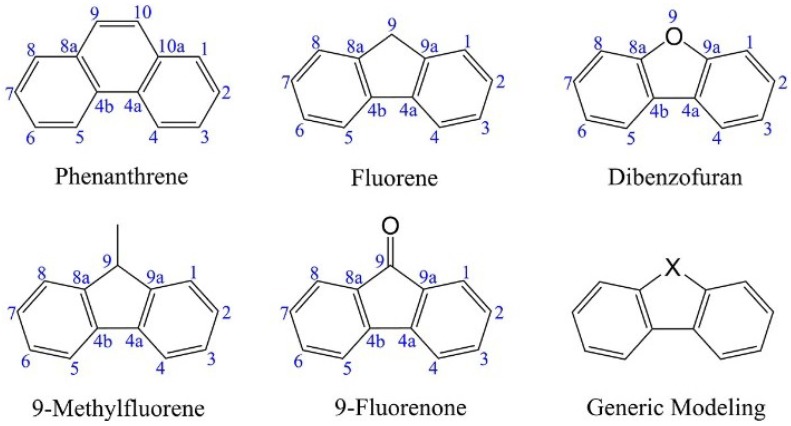
Illustrative structural diagrams for phenanthrene, fluorene, 9-methylfluorene, 9‑fluorenone, dibenzofuran and a generic modeling for them.

The structure formulas of Phe, Flu, 9MF, 9FO and DF are shown in Scheme 1. Those structures show high similarity to each other. Therefore, if a generic modeling is used, Phe will be got while “–X–” stands for “–CH=CH–”, *etc.* The change routes mentioned in this study are mainly focused on breaking the middle ring of these molecules, and then replacing the “–X–” by oxygen atom.

### 2.1. Reaction from Phenanthrene to Dibenzofuran

In a flame, aromatic compounds might be attacked by active radicals, and then be disintegrated [28]. Hydroxyl radical (∙OH) turns out to be one of the dominant chain carriers, and plays an important role in various aspects of combustion chemistry [29,30]. In addition to ∙OH, chlorine monoxide radical (ClO∙) becomes important in the flame of chlorinated organic compounds. The mole fraction of ClO∙ reached 4.01 × 10^−4^, *i.e.*, 1.3 times the concentration of ∙OH, when ammonium perchlorate was used as an oxidizer in a hydrogen-breathing combustion engine [31]. The combustion of CH_3_Cl, CH_2_Cl_2_, and CHCl_3_ in air showed that the mole fractions of ClO∙ radicals under fuel lean conditions gained at least 100 times higher than those of ∙OH radicals at temperatures lower than 900 K [32].

**Figure 1:**
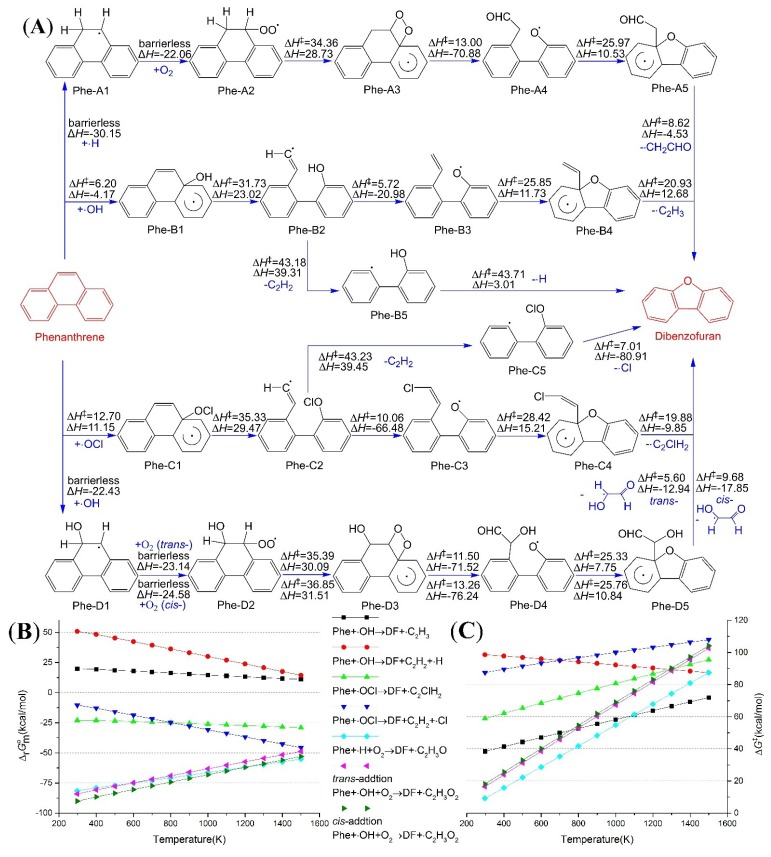
Dibenzofuran formation paths from phenanthrene (Phe); (**A**) Reaction scheme, enthalpies were calculated at 1000 K, and energy unit is kcal/mol; (**B**) Standard Gibbs energy changes for the overall reactions; (**C**) Activation Gibbs energies for the overall reactions (We accumulate the Gibbs energy change of each elementary reaction in turn, and the maximum value in a reaction route at given temperature is selected for the $∆G^{‡}$).

There are 14 carbon atoms with sp^2^ hybridization in the three fused aromatic rings of Phe, and ∙OH radical can add to any one of the carbon atoms. Among all the possible additions, C_8a_ (or C_10a_, they are the equivalent atoms considering symmetry, as shown in Scheme 1) and C_9_ (or C_10_) are chosen as the only reaction sites. Radicals could add at other positions in the Phe molecule, but those are not relevant to the formation of DD and DF and hence they were excluded from mechanistic analysis. The bonding between ∙OH and C_8a_ will finally create DF, and Figure 1A illustrates the predicted reaction route. Activation enthalpies ($∆H^{‡}$), activation Gibbs energies ($∆G^{‡}$), standard enthalpy changes (${∆}_{r}H_{m}^{\text{o}}$) and Gibbs energy changes (${∆}_{r}G_{m}^{\text{o}}$) for every elementary reaction are listed in Tables S1 and S2 (attached as supplementary materials). We calculated the data every 100 K from 1500 to 298.15 K. The bonding reaction between ∙OH and C_8a_ of Phe is an exothermic process, and the reaction enthalpy amounts to –4.17 kcal/mol at 1000 K. The activation enthalpy of this addition reaches as low as 6.20 kcal/mol. The ring-opening reaction of the addition product—Phe-B1—is an endothermic reaction with a potential barrier of 31.73 kcal/mol. The ring-opening seems to be an unfavorable process for the standard Gibbs energy change is 40.41 kcal/mol at 1000 K, although the value is already significantly lower than those of the C_8a_–C_9_ directly dissociation (177.90 kcal/mol). The subsequent H‑migration and C_2_H_3_-leaving reactions eventually produce DF. As a whole, the transformation (Phe + ∙OH → DF + ∙C_2_H_3_) absorbs 22.28 kcal/mol heat at 1000 K. Affected by this, the ${∆}_{r}G_{m}^{\text{o}}$ presents positive within the temperature range from 1500 to 298.15 K, as shown in Figure 1B.

The reaction mechanism of ClO∙ adding to C_8a_ of Phe is similar to that of ∙OH. The five elementary reactions are addition, ring-opening, Cl-migration, cyclization and C_2_ClH_2_-leaving in turn. The ring‑opening and cyclization reactions have higher potential barriers and more absorbed heat than the equivalent elementary reactions in the OH-Phe addition process. However, the Cl-migration and C_2_ClH_2_‑leaving reactions release more heat, and then the enthalpy change for the overall reaction drops into negative, for instance, −20.50 kcal/mol at 1000 K. The ${∆}_{r}G_{m}^{\text{o}}$ drop correspondingly into negative, *i.e.*, about −25.00 kcal/mol in temperature range 1500–298.15 K.

The removal of acetylene is also a possible reaction of Phe-B2, with the exception of H-migration. The reaction enthalpy for this decomposition reaches up to 43.18 kcal/mol. The ${∆}_{r}G_{m}^{\text{o}}$ will drop slowly into negative only if the temperature rises above 1100 K, and this suggests that the removal of acetylene occurs mainly in high temperatures. The subsequent cyclization-dehydrogenation reaction has been proved to be an elementary reaction, the intrinsic reaction coordinate (IRC) analysis of the reaction Phe-B5 → DF + ∙H is displayed on the Figure S1 (attached as supplementary materials). This reaction also has a high potential barrier; therefore, the importance of this reaction channel is watered down again. In Figure 1B, the ${∆}_{r}G_{m}^{\text{o}}$ for the overall reaction (Phe + ∙OH → DF + C_2_H_2_ + ∙H) is always greater than 14.39 kcal/mol. Figure 1C presents the variation tendencies of activation Gibbs energies for the overall reactions along with temperature, and we can see that the activation Gibbs energy always greater than 87.40 kcal/mol. Notwithstanding the removal of acetylene from Phe-C2 is exactly alike to that of Phe-B2, the overall reaction (Phe + ∙OCl → DF + C_2_H_2_ + ∙Cl) has a negative ${∆}_{r}G_{m}^{\text{o}}$ owing to the substantial reduce in Gibbs energy during the cyclization and Cl-leaving reaction.

Adding to C_8a_ of Phe has a chance to create DF, but not a superior route. For example, the reaction enthalpy of ∙OH adding to C_9_ (or C_10_) of Phe amounts to −22.43 kcal/mol at 1000 K, which is obviously lower than the value of adding to C_8a_. Phe-D1 will be produced without any transition state. Similarly, H‑adding reaction also has no potential barrier, and Phe-A1 stands for the H-Phe adduct in Figure 1A. The unpaired electron of Phe-A1 mainly locates in the C_10_, and further oxidation occurs between C_10_ and O_2_, the greatest abundant oxidant in flame. The standard enthalpy change of C–O bonding is −22.06 kcal/mol at 1000 K, and this adding process has no transient state. The outer oxygen atom of Phe-A2 may bond with the adjacent unsaturated carbon atom, and an intermediate—Phe‑A3—with a four-membered ring appears. The elementary reaction is an endothermic process, resulting from the tensile force of the ring. On the other hand, Phe-A3 tends to break the O-O and C-C bonds in the four-membered ring simultaneously, and the driving force of bond breaking is also the tensile force. Phe‑A4 is a more stable state comparing with Phe-A2, and then ${∆}_{r}H_{m}^{\text{o}}$ from Phe-A2 to Phe-A4 reaches −42.15 kcal/mol at 1000 K. DF will be finally formed via cyclization and CH_2_CHO-leaving, which are very similar to the equivalent reactions in the pathway Phe-B. The *cis/trans* isomerism will be found in the reaction process, when O_2_ adds to Phe-D1. The energy data of *trans-*addition are placed above the arrows, in Figure 1A, and the data of *cis-*addition are located under the arrows. The subsequent reactions resemble to reaction pathway Phe-A mentioned above, except for the minor numerical difference.

The standard Gibbs free energy changes for the overall reaction from Phe to DF are calculated and presented in Figure 1B. The activation Gibbs energies for the overall reactions are plotted against temperature and shown in Figure 1C. The detailed data for every elementary reaction are attached in Tables S1 and S2. There is little chance that ∙OH oxides Phe solely into DF, but ClO∙ has more chances. There will be opportunity for O_2_ to take part in and change Phe into DF, once C_9_ of Phe is saturated. The radicals, which can bond with C_9_ and facilitate the combination between the radical-Phe adduct and O_2_, are not restricted to ∙H and ∙OH, *i.e.*, the substituent on C_9_ has a minimal impact on the transformation process. As shown in Figure 1A, the activation enthalpies for each elementary reactions in channel Phe-A and Phe-D are smaller than 37.63 kcal/mol, and $∆G^{‡}$ are smaller than 41.50 kcal/mol within the temperature range 1500–298.15 K. The ${∆}_{r}G_{m}^{\text{o}}$ for overall reactions (Phe + ∙H + O_2_ → DF + ∙C_2_H_3_O and Phe + ∙OH + O_2_ → DF + ∙C_2_H_3_O_2_) are below −48.85 kcal/mol. So the oxidation routes—Phe-A and Phe-D—are superior scenarios than Phe-B and Phe-C.

### 2.2. Reaction from Fluorene to Dibenzofuran and 9-Fluorenone

**Figure 2:**
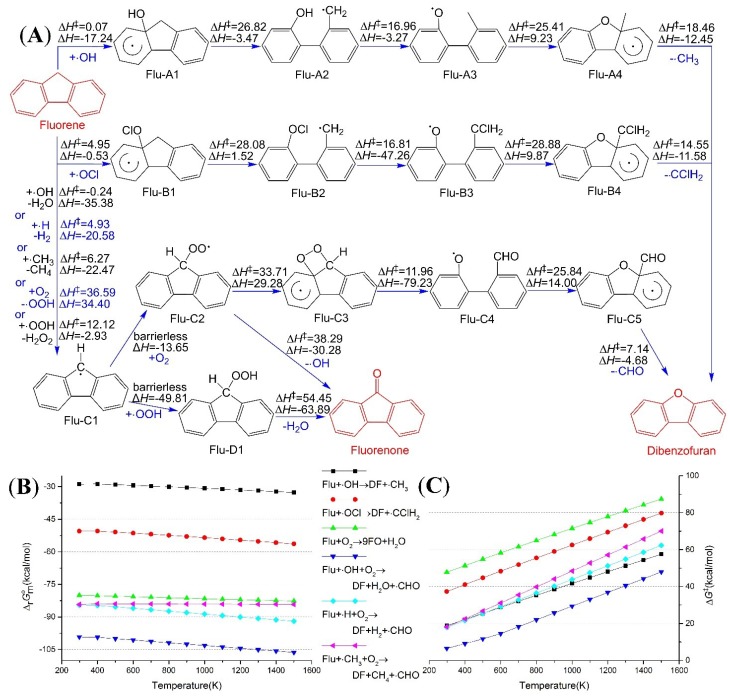
Dibenzofuran and 9-fluorenone formation paths from fluorene (Flu); (**A**) Reaction scheme, enthalpies were calculated at 1000 K, and energy unit is kcal/mol; (**B**) Standard Gibbs energy changes for the overall reactions; (**C**) Activation Gibbs energies for the overall reactions.

Our calculations show that the bond length for C_8a_–C_9_ in a Flu molecule is 1.504 Å, and 1.437 Å is the length in a Phe molecule. The C_8a_–C_9_ bond in Flu is a typical single bond, in comparison with the experimental bond lengths for C–C and C=C (that is, 1.536 Å in ethane and 1.339 Å in ethylene [33]), while the C_8a_–C_9_ bond in Phe appears a hybrid between single and double bond. This difference in bonding properties makes an impact on the breaking of C_8a_–C_9_ bond. The standard enthalpy change for breaking C_8a_–C_9_ bond in Flu amounts to 129.89 kcal/mol at 1000 K, and this is significantly smaller than the energy change for Phe (191.84 kcal/mol). In addition, the activation enthalpy of ∙OH adding to C_8a_ of Flu is 6.13 kcal/mol lower than the value of Phe; and the reaction enthalpy is 13.07 kcal/mol lower than that of Phe at 1000 K. In summary, ∙OH becomes much easier to bond with C_8a_ of Flu, and the same goes for ClO∙ addition. As shown in Figure 2A, the ring-opening reactions of Flu-A1 and Flu-B1 occur much easier, for the bond energy of C_8a_–C_9_ in Flu seems much lower than that in Phe. The subsequent elementary reactions for Phe–B2 are H-migration, cyclization and CH_3_-leaving, while the subsequent elementary reactions for Phe–C2 are Cl–migration, cyclization and CClH_2_-leaving. Throughout the elementary reactions from Flu and ∙OH to DF and ∙CH_3_, the activation enthalpies are always less than 27.38 kcal/mol. The ${∆}_{r}G_{m}^{\text{o}}$ for the overall reaction are between −28.88 and −32.68 kcal/mol within the investigated temperatures. Therefore, the reaction (Flu + ∙OH → DF + ∙CH_3_) is a kinetically and thermodynamically feasible change. The standard Gibbs energy change for the reaction (Flu + ClO∙ → DF + ∙CClH_2_) is at least 21.63 kcal/mol lower than the ${∆}_{r}G_{m}^{\text{o}}$ for Flu + ∙OH → DF + ∙CH_3_, *i.e.*, ClO∙ is much easier to oxidize Flu into DF. Figure 2B,C show the ${∆}_{r}G_{m}^{\text{o}}$ and $∆G^{‡}$ for each overall reaction, respectively. The calculated $∆H^{‡}$, ${∆}_{r}H_{m}^{\text{o}}$, $∆G^{‡}$ and ${∆}_{r}G_{m}^{\text{o}}$ for every elementary reaction are listed in Tables S3 and S4.

Besides addition, H-abstraction is also a feasible route to form DF. Flu has a carbon atom with sp^3^ hybrids unlike most PAHs, and therefore, this atom numbered 9 in Flu cannot participate in delocalized *π* bonding. If one of the hydrogen atoms bonding with C_9_ is dislodged, the hybridized type of C_9_ will be sp^2^, and the unpaired electron will participate in delocalized π bonding accordingly. The H-abstraction reaction take place easily under the influence of delocalization energy. The comparison has been made among the H-abstractions, in which ∙OH, HOO∙, ∙H, ∙CH_3_ and O_2_ are used to eliminate the hydrogen atom bonding with C_9_ of Phe, Flu and 9MF, and the thermodynamic parameters of these reactions at 1000 K are listed in Table 1. The activation enthalpies for the H-abstractions of Flu are always less than those of Phe, additionally reaction enthalpies, activation Gibbs energies and reaction Gibbs energies under normal conditions show the same regularity. This means that Flu is more likely to lose the hydrogen atom than Phe.

The product of H-abstraction—Flu–C1—can be oxidized by oxygen molecule. The first step of oxidation occurs between Flu–C1 and oxygen molecule, and this addition reaction is an exothermic process without a transition state. This bonding creates a peroxide radical—Flu–C2, and then an intermediate with a four-membered ring like dioxetane will be obtained if the outer oxygen atom is linked with C_8a_. Though the formation of this dioxetane-like intermediate is an endothermic process, dioxetane-ring-opening reaction releases a lot of energy, and gives rise to Flu-C4. As the sum total of heat effect from Flu–C2 to Flu–C4, 49.95 kcal/mol of heat will be released. Then the cyclization reaction accompanying with the CHO-leaving reaction generates DF finally.

In addition to DF, 9FO is also the potential oxidation product of Flu. The loss of hydroxyl radical will change Flu–C2 into 9FO. The activation enthalpy and standard enthalpy change for this reaction at 1000 K are 38.29 and −30.28 kcal/mol, respectively. The extent of this decomposition reaction is great, for the ${∆}_{r}G_{m}^{\text{o}}$ at 1000 K reaches −64.05 kcal/mol. Another formation channel of 9FO is the reaction between Flu and O_2_. As mentioned above, one of the hydrogen atoms which bond with C_9_ of Flu can be abstracted by O_2_, and give rise to Flu-C1 and ∙OOH. It is worth mentioning that the coupling of these two radicals is accompanied by the spin-flip of one unpaired electron. There are two unpaired electrons with same direction of spin in O_2_, and when the H-abstraction occurs, the two unpaired electrons dispersed in Flu–C1 and ∙OOH will have the same spin direction. The spin-flip will occur when Flu-C1 and ∙OOH collide with each other, so that the two unpaired electrons can form a covalent bond effectively. The released heat of the spin-flip and bonding process amounts to 49.81 kcal/mol at 1000 K, and this entropy reducing reaction remains to be a spontaneous process below 1200 K, benefiting from reaction heat. The newly generated Flu-D1 has a strong tendency to decompose into 9FO and water, as can be seen from ${∆}_{r}G_{m}^{\text{o}}$, e.g., −100.38 kcal/mol at 1000 K. The overall reaction (Flu + O_2_ → 9FO + H_2_O) is a bimolecular reaction, thus the activation Gibbs energy is higher than those reactions initiated by radicals, as shown in Figure 2C. The formation of 9FO should not be ignored for some reasons, such as that the concentration of O_2_ is much higher than radicals, and that there are only three elementary reactions.

**Table 1:** Comparison of the C_9_-H abstraction reactions at 1000 K (energy unit is kcal/mol).

H-Abstraction Reactions		$∆H^{‡}$	${∆}_{r}H_{m}^{o}$	$∆G^{‡}$	${∆}_{r}G_{m}^{o}$
∙OH	Phenanthrene	3.65	−6.22	28.79	−12.50
	Fluorene	−0.24	−35.38	24.01	−38.48
	9-Methylfluorene	−1.38	−39.37	24.69	−48.04
HOO∙	Phenanthrene	25.86	26.22	53.23	22.81
	Fluorene	12.12	−2.93	44.63	−3.67
	9-Methylfluorene	10.39	−6.92	41.79	−12.73
∙H	Phenanthrene	14.96	8.58	35.54	1.97
	Fluorene	4.93	−20.58	28.27	−24.01
	9-Methylfluorene	3.24	−24.57	25.71	−33.57
∙CH_3_	Phenanthrene	17.26	6.69	45.50	6.58
	Fluorene	6.27	−22.47	43.53	−19.40
	9-Methylfluorene	6.75	−26.46	34.63	−28.96
O_2_	Phenanthrene	58.34	63.55	78.94	54.91
	Fluorene	36.59	34.40	62.90	28.93
	9-Methylfluorene	33.97	30.41	60.21	19.37

### 2.3. Reaction from 9-Methylfluorene to Dibenzofuran and 9-Fluorenone

Under the influence of methyl, the length of C_8a_–C_9_ bond in 9MF is 1.515 Å, which is slightly longer than the length in Flu—1.512 Å. Subsequent oxidation reactions of 9MF take place more easily, as shown in Figure S2. The calculated $∆H^{‡}$, ${∆}_{r}H_{m}^{\text{o}}$, $∆G^{‡}$ and ${∆}_{r}G_{m}^{\text{o}}$ for every elementary reaction are listed in Tables S5 and S6. In general, the elementary reactions of 9MF oxidation have slightly lower energy barriers and release a little more heat than those of Flu oxidations. In order to reduce in symmetry, the process of ∙OH and ClO∙ adding to 9MF have *cis-trans* isomers, as shown in Figure S2A. There are significant differences in forming 9FO: 9MF-C2 cannot eliminate CH_3_O∙ directly, and CH_3_OH cannot break away from 9MF-D1. In other words, 9FO will be formed when ∙OH and ∙CH_3_ depart from 9MF-D1 in turn. The elementary reaction, which ∙OH separates itself from 9MF-D1, breaks the O–O bond, and therefore it is a barrierless process with the gradually increasing enthalpy. The reaction enthalpy of OH-leaving reaches 44.11 kcal/mol at 1000 K. The CH_3_-leaving is relatively easy to accomplish, for the activation enthalpy is only 10.98 kcal/mol, and the reaction Gibbs energy is −36.84 kcal/mol at 1000 K.

### 2.4. Reaction from 9-Fluorenone to Dibenzofuran

**Figure 3:**
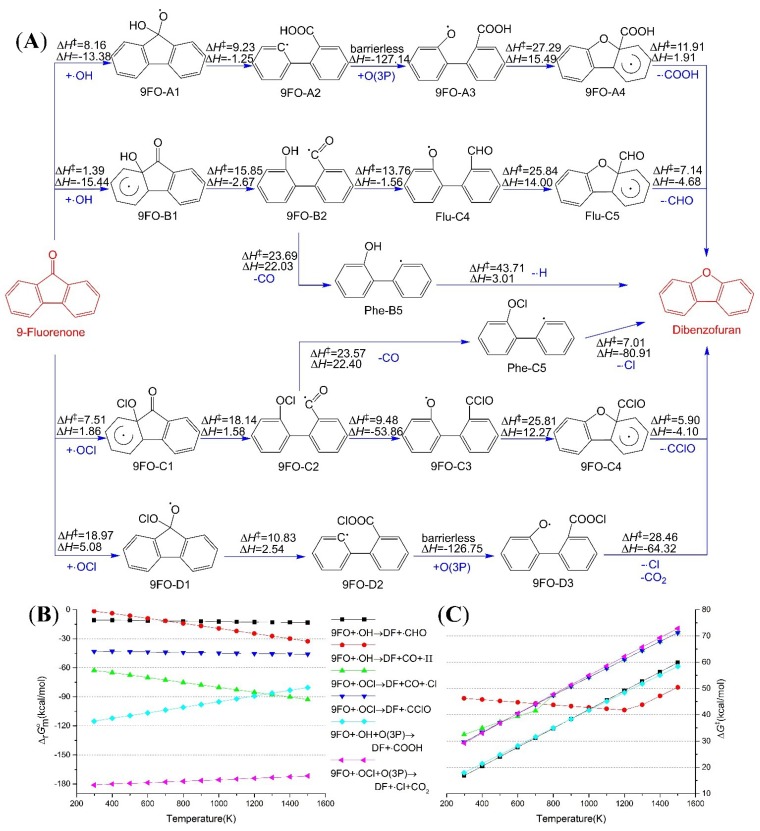
Dibenzofuran formation paths from 9-fluorenone (9FO); (**A**) Reaction scheme, enthalpies were calculated at 1000 K, and energy unit is kcal/mol; (**B**) Standard Gibbs energy changes for the overall reactions; (**C**) Activation Gibbs energies for the overall reactions.

The intermediate 9FO-B1 will be achieved if ∙OH adds to C_8a_ of 9FO, as shown in Figure 3A. This elementary reaction has a low activation enthalpy (1.39 kcal/mol), and the releases heat is 15.44 kcal/mol at 1000 K. The ring-opening reaction of 9FO–B1 is easy to complete in comparison with the equivalent reactions of Flu and 9MF, because the activation enthalpy is significantly decreased (15.85 kcal/mol for 9FO, 26.82 kcal/mol for Flu, and 23.14 kcal/mol for 9MF). There are two possible reaction pathways for 9FO–B2: (I) the H-migration creates Flu–C4 (an intermediate mentioned in the transformation route from Flu to DF); (II) carbon monoxide may slip the leash and leaves Phe–C5 alone. Each of the competing reactions has its advantages and disadvantages. When activation enthalpy and reaction enthalpy are taken into account, the change from 9FO–B2 to Flu–C4 will be favorable; but in terms of standard reaction Gibbs energy, Phe–C5 and CO are the dominant products. The point of view we take is that Phe–C5 is more favorable at higher temperatures and Flu–C4 at lower temperatures. The calculated $∆H^{‡}$, ${∆}_{r}H_{m}^{\text{o}}$, $∆G^{‡}$ and ${∆}_{r}G_{m}^{\text{o}}$ for every elementary reaction at different temperatures are listed in Tables S7 and S8.

So take the other case, ∙OH bonds with C_9_ of 9FO. The first elementary reaction is to form a C–O bond. The bonding process has a low activation enthalpy—8.16 kcal/mol, and the released heat is 13.38 kcal/mol at 1000 K. The subsequent reaction is the breakage of C_8a_–C_9_ bond, and the activation enthalpy of this breakage—9.23 kcal/mol—is lowest among all the cases mentioned in this study. Moreover, then there are several routes to change 9FO-A2 into DF, such as O(3P) adding to 9FO–A2 (as shown in Figure 3A), O_2_ adding to 9FO–A2 and H-migration of 9FO-A2 (Figure S3A). In the case of O(3P) adding, the standard Gibbs energy changes for the overall reaction are significantly lower than those of ∙OH adding to C_8a_ at any temperature between 1500 and 298.15 K, as shown in Figure 3B.

The reaction mechanism of ClO∙ addition is similar to those of ∙OH addition. The bonding between ClO∙ and C_8a_ and the breaking of C_8a_–C_9_ bond will be a little more difficult to occur than the reactions of ∙OH, when thought of a little higher activation enthalpies and reaction enthalpies. However, the Cl-migration reaction is much more feasible than H-migration. Under standard conditions and 1000 K, the activation enthalpies for Cl- and H-migration are 9.48 and 13.76 kcal/mol, respectively; the reaction enthalpies are −53.86 and −1.56 kcal/mol; the activation Gibbs energies are 13.96 and 22.25 kcal/mol; and the reaction Gibbs energies are −51.45 and 1.05 kcal/mol. This means that the superiority of the ClO∙ addition exists similarly in the DF formation from 9FO. In the reaction channel of ClO∙ adding to C_9_, the possible reactions of intermediate 9FO-D2 are O(3P)addition (as shown in Figure 3A), O_2_ addition and Cl-migration (Figure S3B). The ${∆}_{r}G_{m}^{\text{o}}\;$ of the overall reaction (9FO + ∙OCl + O(3P) → DF + CO_2_ + ∙Cl) is lower than −171.73 kcal/mol, and such a low Gibbs energy change shows that the reaction extant is great.

## 3. Experimental Section

The high-accuracy quantum chemical calculations were performed with Gaussian 09 programs [34], using the method based on the density functional theory including dispersion correction (DFT-D). DFT-D is an atomic pairwise correction that is added to the result of a converged standard DFT calculation [35]. The D3 version of Grimme’s dispersion (DFT-D3) has been refined regarding higher accuracy, broader range of applicability, and less empiricism [36,37]. As one of the DFT-D3 methods, the M06‑2X‑GD3 provides anisotropy of van der Waals interactions that agrees best with both higher level of theory and experiments [38,39]._ENREF_24 All the geometrical conformations and vibration parameters were calculated on the M06‑2X‑GD3/6-311+G(d,p) level using Gaussian 09. In addition, the corrected factors of enthalpies and Gibbs energies (*H*_CF_, *G*_CF_) were also acquired through the Freq (vibrational frequencies) calculations on the M06‑2X‑GD3/6-311+G(d,p) level, by means of specifying the temperature at which substances inhabit. These calculations carried out at every 100 K from 1500 to 298.15 K. The M06‑2X‑GD3/aug-cc-pVQZ was employed to calculate the single point energies (*E*_SP_) of various species. The final enthalpies (*H*) at 1000 K were worked out by the expression—*H*_1000 K_ = *E*_SP_ + *H*_CF, 1000 K_. Analogously, all the *H* and *G* at different temperature were figured out. Each transition state was verified to connect the related reactants and products by performing an intrinsic reaction coordinate (IRC) analysis.

## 4. Conclusions

Calculations show that Phe, Flu, 9MF and 9FO can be oxidized into DF by ∙OH, ClO∙, O_2_ or O(3P) in flame or flue gas. The C_8a_–C_9_ bond could be broken when ∙OH or ClO∙ adds to C_8a_ of these molecules. The middle ring is broken most easily by 9FO, followed by 9MF, Flu, and, lastly, Phe. In general, the addition and ring-opening reactions of ClO∙ are a little more difficult to accomplish compared with those of ∙OH, but the Gibbs energy changes of the overall reactions show that the ClO∙ addition are dominant channels for the ${∆}_{r}G_{m}^{\text{o}}$ are at least 21.63 kcal/mol lower than the value for the equivalent reactions initiated by ∙OH.

Adding O_2_ to C_10_ of the radical-Phe adduct, whose C_9_ has been saturated by ∙H or ∙OH, is a more feasible oxidation route than the channel Phe-B (Phe + ∙OH → DF + ∙C_2_H_3_). Formation paths of 9FO are explored during the oxidation of Flu and 9MF, and the results show that the extents of producing 9FO are great, and yet the energy barriers are higher than those of creating DF. DF may also stem from the fact that ∙OH or ClO∙ adds to the C_9_ of 9FO, and it is worth mentioning that (I) an additional oxidant—molecular or atomic oxygen—is needed and (II) more heat is released in this reaction route.

## Supplementary Materials

Supplementary materials can be found at http://www.mdpi.com/1422-0067/16/03/5271/s1.
