# Supplementary material for: Mechanistic Studies on the Dibenzofuran Formation from Phenanthrene, Fluorene and 9–Fluorenone
Source: Int J Mol Sci. 2015 Mar 6;16(3):5271–84. doi: 10.3390/ijms16035271 (PMC4394475; doi:10.3390/ijms16035271)
Supplement: Supplementary file 1 [file ijms-16-05271-s001.pdf]

Supplementary Information

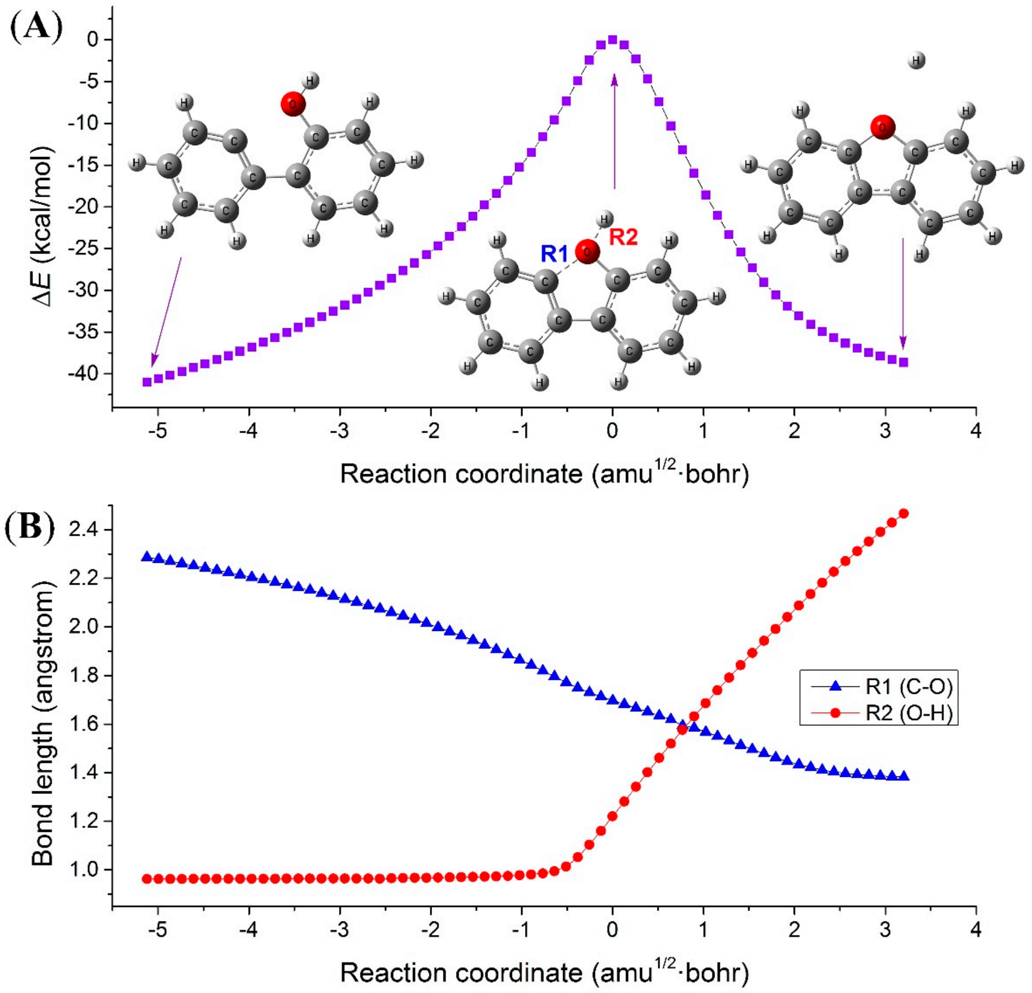

Figure S1. Intrinsic reaction coordinate (IRC) analysis of the reaction Phe-B5→DF + ·H; (**A**) The energy changes along with the reaction coordinate; (**B**) The changes of bond lengths along with the reaction coordinate.

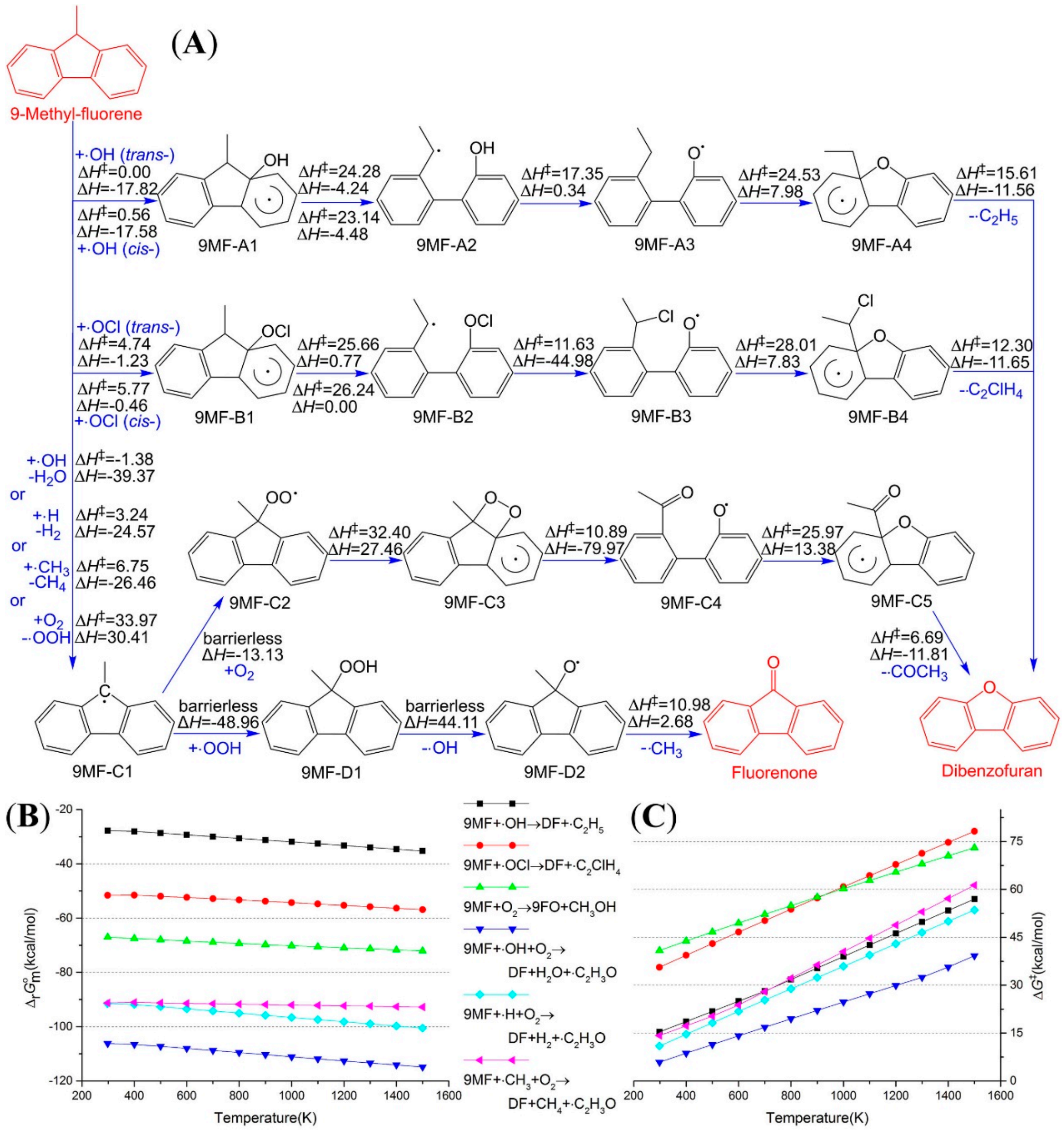

Figure S2. Dibenzofuran and 9-fluorenone formation paths from 9-methylfluorene (9MF); (**A**) Reaction scheme, enthalpies is were calculated at 1000 K, and energy unit is kcal/mol; (**B**) Standard Gibbs energy changes for the overall reactions; (**C**) Activation Gibbs energies for the overall reactions.

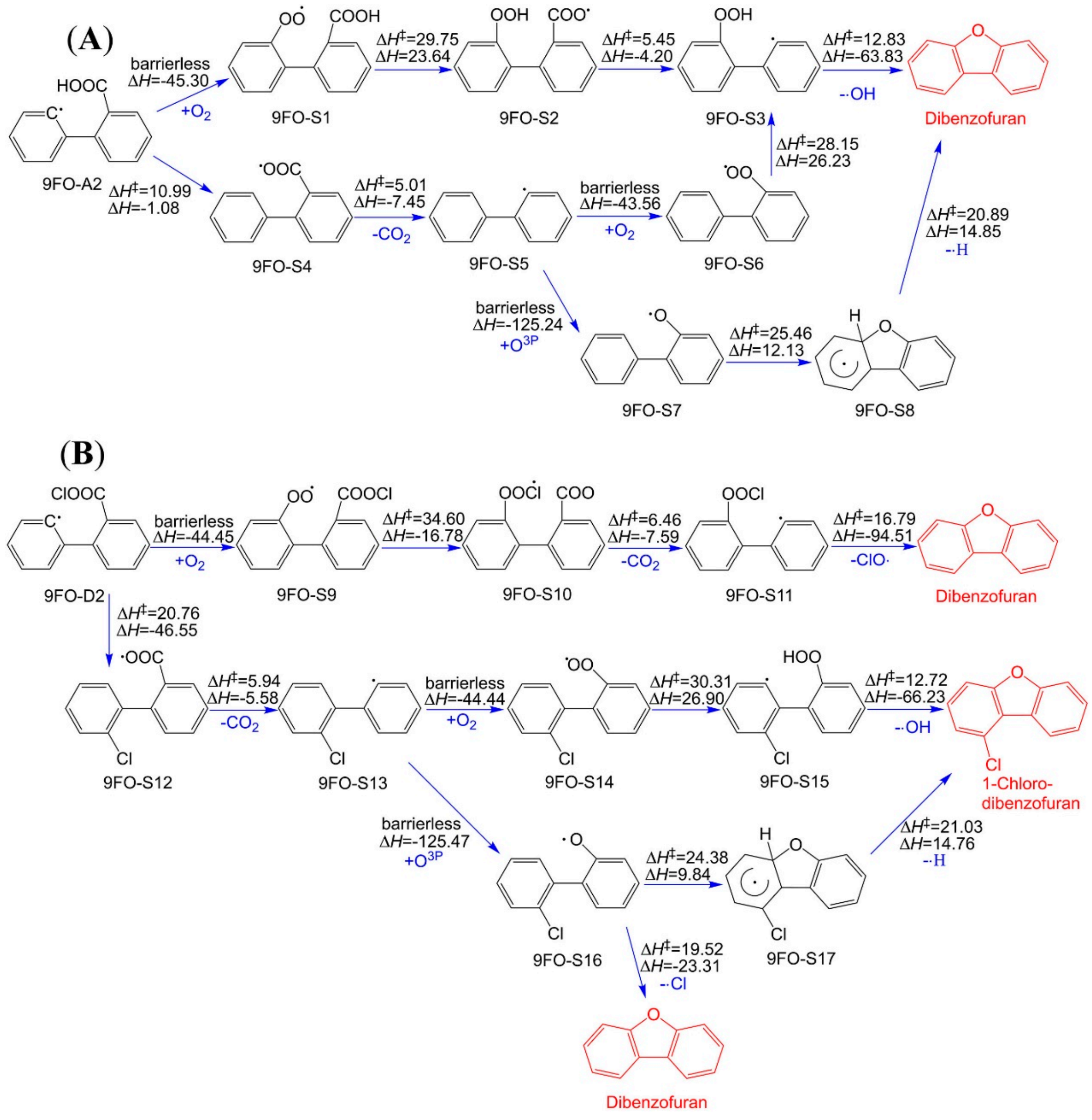

Figure S3. (**A**) Supplementary reaction channels for 9FO-A2 (9FO stands for 9-fluorenone); (**B**) Supplementary reaction channels for 9FO-D2.

Table S1. Calculated activation enthalpies and standard enthalpy changes (in kcal/mol) for the reactions from phenanthrene (Phe) to dibenzofuran (DF).

No.	Elementary Reactions		Temperature (K)												
			**298.15**	**400**	**500**	**600**	**700**	**800**	**900**	**1000**	**1100**	**1200**	**1300**	**1400**	**1500**
1	Phe + ·H → Phe-A1	$\Delta_r H_m^o$	−28.99	−29.27	−29.50	−29.69	−29.84	−29.97	−30.07	−30.15	−30.21	−30.26	−30.28	−30.30	−30.30
2	Phe-A1 + O_2 → Phe-A2	$\Delta_r H_m^o$	−22.79	−22.86	−22.85	−22.76	−22.63	−22.47	−22.27	−22.06	−21.84	−21.60	−21.36	−21.11	−20.85
3	Phe-A2 → Phe-A3	$\Delta H^{\ddagger}$	35.22	35.12	35.01	34.90	34.78	34.65	34.51	34.36	34.21	34.05	33.88	33.72	33.55
		$\Delta_r H_m^o$	28.38	28.39	28.43	28.50	28.56	28.62	28.68	28.73	28.78	28.82	28.86	28.90	28.93
4	Phe-A3 → Phe-A4	$\Delta H^{\ddagger}$	13.72	13.70	13.64	13.55	13.43	13.30	13.16	13.00	12.84	12.67	12.50	12.32	12.14
		$\Delta_r H_m^o$	−71.40	−71.17	−71.03	−70.95	−70.91	−70.89	−70.88	−70.88	−70.88	−70.89	−70.89	−70.90	−70.91
5	Phe-A4 → Phe-A5	$\Delta H^{\ddagger}$	27.16	26.98	26.82	26.65	26.49	26.32	26.15	25.97	25.79	25.61	25.42	25.24	25.05
		$\Delta_r H_m^o$	10.48	10.43	10.43	10.44	10.46	10.49	10.51	10.53	10.55	10.57	10.59	10.60	10.61
6	Phe-A5 → DF + ·C_2H_3O	$\Delta H^{\ddagger}$	9.59	9.51	9.40	9.27	9.12	8.96	8.79	8.62	8.43	8.25	8.06	7.87	7.67
		$\Delta_r H_m^o$	−2.43	−2.60	−2.85	−3.14	−3.46	−3.80	−4.16	−4.53	−4.90	−5.29	−5.67	−6.07	−6.46
7	Phe + ·OH → Phe-B1	$\Delta H^{\ddagger}$	5.77	5.77	5.81	5.87	5.94	6.02	6.11	6.20	6.30	6.39	6.49	6.58	6.68
		$\Delta_r H_m^o$	−5.57	−5.52	−5.38	−5.19	−4.96	−4.71	−4.44	−4.17	−3.89	−3.61	−3.32	−3.03	−2.74
8	Phe-B1 → Phe-B2	$\Delta H^{\ddagger}$	32.25	32.29	32.27	32.21	32.12	32.00	31.87	31.73	31.58	31.42	31.26	31.09	30.92
		$\Delta_r H_m^o$	22.43	22.62	22.75	22.85	22.91	22.96	23.00	23.02	23.04	23.06	23.08	23.09	23.10
9	Phe-B2 → Phe-B3	$\Delta H^{\ddagger}$	6.62	6.39	6.21	6.07	5.96	5.87	5.79	5.72	5.65	5.57	5.49	5.41	5.32
		$\Delta_r H_m^o$	−20.41	−20.55	−20.67	−20.77	−20.84	−20.90	−20.95	−20.98	−21.00	−21.01	−21.02	−21.02	−21.02
10	Phe-B3 → Phe-B4	$\Delta H^{\ddagger}$	27.05	26.87	26.70	26.54	26.37	26.20	26.03	25.85	25.67	25.49	25.31	25.12	24.93
		$\Delta_r H_m^o$	11.74	11.67	11.65	11.66	11.68	11.69	11.71	11.73	11.75	11.76	11.78	11.79	11.80
11	Phe-B4 → DF + ·C_2H_3	$\Delta H^{\ddagger}$	21.58	21.60	21.56	21.47	21.36	21.23	21.09	20.93	20.77	20.60	20.43	20.25	20.07
		$\Delta_r H_m^o$	14.71	14.57	14.34	14.06	13.74	13.40	13.04	12.68	12.30	11.93	11.55	11.16	10.78
12	Phe-B2 → Phe-B5 + C_2H_2	$\Delta H^{\ddagger}$	43.23	43.38	43.46	43.49	43.46	43.40	43.30	43.18	43.05	42.89	42.73	42.56	42.39
		$\Delta_r H_m^o$	40.13	40.18	40.15	40.07	39.93	39.75	39.54	39.31	39.06	38.79	38.52	38.23	37.95
13	Phe-B5 → DF + ·H	$\Delta H^{\ddagger}$	43.96	43.88	43.83	43.80	43.78	43.76	43.73	43.71	43.67	43.63	43.58	43.52	43.45
		$\Delta_r H_m^o$	2.51	2.59	2.66	2.73	2.81	2.88	2.95	3.01	3.07	3.12	3.16	3.19	3.21
14	Phe + ·OCl → Phe-C1	$\Delta H^{\ddagger}$	11.69	11.83	11.99	12.14	12.29	12.43	12.56	12.70	12.82	12.95	13.07	13.18	13.30
		$\Delta_r H_m^o$	8.89	9.16	9.47	9.80	10.14	10.48	10.82	11.15	11.48	11.81	12.13	12.46	12.77

Table S1. *Cont.*

No.	Elementary Reactions		Temperature (K)												
			**298.15**	**400**	**500**	**600**	**700**	**800**	**900**	**1000**	**1100**	**1200**	**1300**	**1400**	**1500**
15	Phe-C1 → Phe-C2	$\Delta H^{\ddagger}$	35.94	35.95	35.91	35.84	35.73	35.61	35.48	35.33	35.18	35.02	34.85	34.68	34.51
		$\Delta_r H_m^o$	28.86	29.05	29.18	29.28	29.35	29.40	29.44	29.47	29.50	29.52	29.54	29.55	29.57
16	Phe-C2 → Phe-C3	$\Delta H^{\ddagger}$	11.25	11.09	10.93	10.76	10.59	10.42	10.24	10.06	9.87	9.69	9.50	9.31	9.12
		$\Delta_r H_m^o$	−65.91	−66.04	−66.15	−66.25	−66.32	−66.39	−66.44	−66.48	−66.52	−66.55	−66.58	−66.61	−66.63
17	Phe-C3 → Phe-C4	$\Delta H^{\ddagger}$	29.63	29.45	29.28	29.11	28.95	28.77	28.60	28.42	28.24	28.06	27.87	27.68	27.49
		$\Delta_r H_m^o$	15.19	15.14	15.13	15.14	15.15	15.17	15.19	15.21	15.23	15.25	15.26	15.27	15.29
18	Phe-C4 → DF + $\cdot C_2ClH_2$	$\Delta H^{\ddagger}$	20.63	20.62	20.56	20.46	20.34	20.20	20.04	19.88	19.71	19.53	19.36	19.17	18.99
		$\Delta_r H_m^o$	−8.42	−8.59	−8.79	−9.00	−9.21	−9.42	−9.64	−9.85	−10.07	−10.28	−10.49	−10.71	−10.92
19	Phe-C2 → Phe-C5 + C_2H_2	$\Delta H^{\ddagger}$	43.28	43.43	43.51	43.54	43.51	43.45	43.35	43.23	43.10	42.95	42.78	42.61	42.44
		$\Delta_r H_m^o$	40.27	40.32	40.29	40.21	40.07	39.89	39.68	39.45	39.20	38.93	38.66	38.38	38.09
20	Phe-C5 → DF + ·Cl	$\Delta H^{\ddagger}$	8.08	7.97	7.84	7.69	7.53	7.36	7.19	7.01	6.82	6.63	6.45	6.26	6.06
		$\Delta_r H_m^o$	−79.62	−79.83	−80.04	−80.24	−80.42	−80.60	−80.76	−80.91	−81.06	−81.20	−81.34	−81.47	−81.60
21	Phe + ·OH → Phe-D1	$\Delta_r H_m^o$	−23.45	−23.46	−23.40	−23.27	−23.10	−22.90	−22.68	−22.43	−22.18	−21.92	−21.65	−21.38	−21.10
22	Phe-D1 + O_2 → *trans*-Phe-D2	$\Delta_r H_m^o$	−23.85	−23.93	−23.92	−23.84	−23.71	−23.54	−23.35	−23.14	−22.91	−22.68	−22.43	−22.18	−21.92
23	*trans*-Phe-D2 → *trans*-Phe-D3	$\Delta H^{\ddagger}$	36.25	36.15	36.04	35.93	35.80	35.67	35.54	35.39	35.24	35.08	34.92	34.76	34.59
		$\Delta_r H_m^o$	29.72	29.73	29.78	29.84	29.91	29.98	30.04	30.09	30.14	30.19	30.23	30.27	30.30
24	*trans*-Phe-D3 → *trans*-Phe-D4	$\Delta H^{\ddagger}$	12.20	12.17	12.12	12.03	11.92	11.79	11.65	11.50	11.34	11.17	10.99	10.82	10.64
		$\Delta_r H_m^o$	−72.12	−71.87	−71.72	−71.63	−71.58	−71.55	−71.53	−71.52	−71.52	−71.52	−71.52	−71.52	−71.52
25	*trans*-Phe-D4 → *trans*-Phe-D5	$\Delta H^{\ddagger}$	26.51	26.34	26.18	26.01	25.85	25.68	25.51	25.33	25.16	24.97	24.79	24.60	24.42
		$\Delta_r H_m^o$	7.78	7.71	7.69	7.69	7.70	7.72	7.73	7.75	7.76	7.77	7.79	7.80	7.80
26	*trans*-Phe-D5 → DF + $\cdot C_2H_3O_2$	$\Delta H^{\ddagger}$	6.74	6.63	6.49	6.33	6.16	5.98	5.79	5.60	5.41	5.21	5.02	4.82	4.62
		$\Delta_r H_m^o$	−10.27	−10.57	−10.92	−11.31	−11.71	−12.11	−12.52	−12.94	−13.35	−13.76	−14.17	−14.59	−15.00
27	Phe-D1 + O_2 → *cis*-Phe-D2	$\Delta_r H_m^o$	−25.24	−25.34	−25.34	−25.27	−25.14	−24.98	−24.79	−24.58	−24.35	−24.11	−23.87	−23.61	−23.35
28	*cis*-Phe-D2 → *cis*-Phe-D3	$\Delta H^{\ddagger}$	37.63	37.55	37.46	37.36	37.25	37.12	36.99	36.85	36.70	36.54	36.38	36.22	36.05
		$\Delta_r H_m^o$	31.08	31.11	31.17	31.24	31.32	31.39	31.45	31.51	31.56	31.60	31.64	31.68	31.71
29	*cis*-Phe-D3 → *cis*-Phe-D4	$\Delta_r H_m^o$	13.97	13.95	13.89	13.80	13.69	13.56	13.41	13.26	13.10	12.93	12.75	12.57	12.39
		$\Delta_r H_m^o$	−76.63	−76.43	−76.32	−76.26	−76.24	−76.23	−76.23	−76.24	−76.25	−76.26	−76.27	−76.28	−76.29

Table S1. *Cont.*

No.	Elementary Reactions		Temperature (K) 298.15	400	500	600	700	800	900	1000	1100	1200	1300	1400	1500
30	*cis*-Phe-D4 → *cis*-Phe-D5	$\Delta H^{\ddagger}$	26.90	26.73	26.58	26.43	26.27	26.10	25.93	25.76	25.58	25.40	25.22	25.04	24.85
		$\Delta_r H_m^o$	10.78	10.72	10.72	10.74	10.76	10.79	10.81	10.84	10.86	10.88	10.90	10.92	10.93
31	*cis*-Phe-D5 → DF + $\cdot C_2H_3O_2$	$\Delta H^{\ddagger}$	10.74	10.66	10.54	10.39	10.23	10.05	9.87	9.68	9.49	9.30	9.11	8.91	8.71
		$\Delta_r H_m^o$	−14.99	−15.33	−15.73	−16.14	−16.57	−17.00	−17.42	−17.85	−18.28	−18.70	−19.13	−19.55	−19.96

Table S2. Calculated Gibbs energies of activation and standard Gibbs energy changes (in kcal/mol) for the reactions from phenanthrene (Phe) to dibenzofuran (DF).

No.	Elementary Reactions		Temperature (K) 298.15	400	500	600	700	800	900	1000	1100	1200	1300	1400	1500
1	Phe + ·H → Phe-A1	$\Delta_r G_m^o$	−23.05	−20.97	−18.87	−16.72	−14.55	−12.35	−10.15	−7.93	−5.70	−3.47	−1.24	1.00	3.23
2	Phe-A1 + O_2 → Phe-A2	$\Delta_r G_m^o$	−10.58	−6.39	−2.28	1.83	5.92	9.99	14.03	18.05	22.05	26.03	29.99	33.94	37.86
3	Phe-A2 → Phe-A3	$\Delta G^{\ddagger}$	36.14	36.47	36.82	37.20	37.59	38.00	38.43	38.87	39.33	39.80	40.29	40.79	41.30
		$\Delta_r G_m^o$	29.09	29.33	29.56	29.78	29.99	30.19	30.38	30.57	30.75	30.92	31.10	31.27	31.44
4	Phe-A3 → Phe-A4	$\Delta G^{\ddagger}$	13.75	13.76	13.78	13.81	13.87	13.94	14.02	14.13	14.25	14.39	14.53	14.70	14.87
		$\Delta_r G_m^o$	−73.90	−74.79	−75.71	−76.66	−77.62	−78.58	−79.54	−80.50	−81.46	−82.42	−83.38	−84.34	−85.30
5	Phe-A4 → Phe-A5	$\Delta G^{\ddagger}$	27.94	28.24	28.57	28.94	29.33	29.75	30.19	30.65	31.13	31.62	32.13	32.65	33.19
		$\Delta_r G_m^o$	11.46	11.81	12.15	12.50	12.84	13.17	13.51	13.84	14.17	14.50	14.82	15.15	15.47
6	Phe-A5 → DF + $\cdot C_2H_3O$	$\Delta G^{\ddagger}$	10.12	10.32	10.53	10.77	11.03	11.31	11.62	11.94	12.28	12.64	13.01	13.40	13.80
		$\Delta_r G_m^o$	−14.53	−18.07	−21.92	−25.71	−29.45	−33.14	−36.78	−40.39	−43.96	−47.49	−50.99	−54.46	−57.91
7	Phe + ·OH → Phe-B1	$\Delta G^{\ddagger}$	14.95	18.08	21.16	24.22	27.27	30.32	33.35	36.37	39.38	42.39	45.38	48.37	51.35
		$\Delta_r G_m^o$	4.00	7.27	10.46	13.61	16.72	19.80	22.84	25.86	28.85	31.82	34.76	37.67	40.57
8	Phe-B1 → Phe-B2	$\Delta G^{\ddagger}$	31.68	31.47	31.27	31.08	30.89	30.73	30.57	30.44	30.32	30.21	30.11	30.03	29.96
		$\Delta_r G_m^o$	20.28	19.52	18.72	17.91	17.08	16.24	15.40	14.55	13.70	12.85	12.00	11.15	10.30
9	Phe-B2 → Phe-B3	$\Delta G^{\ddagger}$	8.55	9.25	9.98	10.75	11.54	12.34	13.15	13.98	14.81	15.64	16.48	17.33	18.19
		$\Delta_r G_m^o$	−20.29	−20.23	−20.13	−20.01	−19.88	−19.74	−19.59	−19.44	−19.28	−19.13	−18.97	−18.81	−18.66
10	Phe-B3 → Phe-B4	$\Delta_r G_m^o$	28.93	29.60	30.30	31.04	31.80	32.59	33.40	34.23	35.08	35.94	36.82	37.71	38.62
		$\Delta_r G_m^o$	13.46	14.06	14.66	15.26	15.86	16.46	17.05	17.65	18.24	18.82	19.41	20.00	20.59

Table S2. *Cont.*

No.	Elementary Reactions		Temperature (K)												
			**298.15**	**400**	**500**	**600**	**700**	**800**	**900**	**1000**	**1100**	**1200**	**1300**	**1400**	**1500**
11	Phe-B4 → DF + $\cdot C_2H_3$	$\Delta G^{\ddagger}$	20.94	20.71	20.50	20.29	20.10	19.93	19.77	19.64	19.52	19.41	19.32	19.24	19.17
		$\Delta_r G_m^o$	2.44	−1.17	−5.10	−8.96	−12.77	−16.54	−20.26	−23.94	−27.58	−31.19	−34.77	−38.32	−41.84
12	Phe-B2 → Phe-B5 + C_2H_2	$\Delta G^{\ddagger}$	41.77	41.24	40.69	40.14	39.58	39.03	38.49	37.96	37.44	36.94	36.45	35.97	35.51
		$\Delta_r G_m^o$	29.29	25.57	21.92	18.29	14.67	11.07	7.49	3.95	0.42	−3.08	−6.56	−10.01	−13.45
13	Phe-B5 → DF + ·H	$\Delta G^{\ddagger}$	45.04	45.43	45.82	46.22	46.63	47.03	47.44	47.86	48.27	48.69	49.12	49.55	49.98
		$\Delta_r G_m^o$	−2.81	−4.07	−5.76	−7.45	−9.15	−10.86	−12.59	−14.31	−16.05	−17.79	−19.53	−21.28	−23.03
14	Phe + ·Ocl → Phe-C1	$\Delta G^{\ddagger}$	22.30	25.90	29.40	32.87	36.32	39.74	43.14	46.54	49.91	53.28	56.64	59.98	63.32
		$\Delta_r G_m^o$	19.57	23.19	26.66	30.07	33.42	36.72	39.98	43.20	46.39	49.55	52.68	55.79	58.87
15	Phe-C1 → Phe-C2	$\Delta G^{\ddagger}$	35.60	35.48	35.37	35.26	35.18	35.10	35.05	35.01	34.98	34.97	34.98	34.99	35.02
		$\Delta_r G_m^o$	25.86	24.81	23.73	22.63	21.52	20.40	19.27	18.14	17.01	15.87	14.73	13.59	12.45
16	Phe-C2 → Phe-C3	$\Delta G^{\ddagger}$	13.42	14.18	14.98	15.80	16.66	17.53	18.43	19.36	20.29	21.25	22.22	23.21	24.21
		$\Delta_r G_m^o$	−65.29	−65.05	−64.79	−64.51	−64.22	−63.91	−63.60	−63.28	−62.96	−62.63	−62.31	−61.98	−61.65
17	Phe-C3 → Phe-C4	$\Delta G^{\ddagger}$	31.53	32.21	32.93	33.67	34.44	35.24	36.06	36.90	37.76	38.63	39.52	40.42	41.34
		$\Delta_r G_m^o$	16.87	17.45	18.03	18.61	19.19	19.76	20.33	20.90	21.47	22.03	22.60	23.16	23.73
18	Phe-C4 → DF + $\cdot C_2ClH_2$	$\Delta G^{\ddagger}$	19.78	19.49	19.21	18.95	18.70	18.48	18.27	18.08	17.91	17.76	17.62	17.49	17.38
		$\Delta_r G_m^o$	−20.03	−23.41	−27.10	−30.75	−34.36	−37.93	−41.48	−45.01	−48.52	−52.00	−55.47	−58.92	−62.36
19	Phe-C2 → Phe-C5 + C_2H_2	$\Delta G^{\ddagger}$	42.01	41.55	41.07	40.58	40.09	39.61	39.13	38.67	38.22	37.78	37.36	36.95	36.55
		$\Delta_r G_m^o$	29.72	26.11	22.55	19.02	15.49	11.99	8.52	5.07	1.64	−1.76	−5.14	−8.50	−11.84
20	Phe-C5 → DF + ·Cl	$\Delta G^{\ddagger}$	8.05	8.06	8.09	8.16	8.25	8.37	8.50	8.66	8.82	9.02	9.23	9.45	9.68
		$\Delta_r G_m^o$	−85.58	−87.02	−88.80	−90.54	−92.24	−93.91	−95.57	−97.21	−98.83	−100.44	−102.03	−103.62	−105.20
21	Phe + ·OH → Phe-D1	$\Delta_r G_m^o$	−14.44	−11.36	−8.34	−5.34	−2.37	0.58	3.50	6.40	9.27	12.12	14.94	17.75	20.53
22	Phe-D1 + O_2 → *trans*-Phe-D2	$\Delta_r G_m^o$	−11.86	−7.75	−3.71	0.33	4.34	8.34	12.32	16.27	20.20	24.11	28.00	31.86	35.72
23	*trans*-Phe-D2 → *trans*-Phe-D3	$\Delta G^{\ddagger}$	36.92	37.16	37.43	37.72	38.03	38.35	38.70	39.06	39.43	39.82	40.22	40.63	41.06
		$\Delta_r G_m^o$	30.44	30.69	30.92	31.14	31.35	31.56	31.75	31.94	32.12	32.30	32.47	32.64	32.81
24	*trans*-Phe-D3 → *trans*-Phe-D4	$\Delta G^{\ddagger}$	12.26	12.29	12.32	12.37	12.44	12.52	12.62	12.73	12.86	13.01	13.17	13.34	13.53
		$\Delta_r G_m^o$	−74.16	−74.90	−75.67	−76.47	−77.29	−78.10	−78.93	−79.75	−80.57	−81.39	−82.21	−83.04	−83.86

Table S2. *Cont.*

No.	Elementary Reactions		Temperature (K)												
			**298.15**	**400**	**500**	**600**	**700**	**800**	**900**	**1000**	**1100**	**1200**	**1300**	**1400**	**1500**
25	*trans*-Phe-D4 → *trans*-Phe-D5	$\Delta G^{\ddagger}$	27.97	28.49	29.05	29.64	30.26	30.90	31.56	32.24	32.94	33.66	34.39	35.14	35.90
		$\Delta_r G_m^o$	9.00	9.43	9.87	10.30	10.74	11.17	11.60	12.03	12.46	12.88	13.31	13.73	14.16
26	*trans*-Phe-D5 → DF + $\cdot C_2H_3O_2$	$\Delta G^{\ddagger}$	6.90	6.97	7.06	7.19	7.35	7.53	7.74	7.96	8.21	8.47	8.75	9.04	9.35
		$\Delta_r G_m^o$	−23.08	−26.84	−30.89	−34.84	−38.73	−42.57	−46.35	−50.08	−53.78	−57.44	−61.06	−64.65	−68.21
27	Phe-D1 + O_2 → *cis*-Phe-D2	$\Delta_r G_m^o$	−12.78	−8.51	−4.30	−0.09	4.09	8.26	12.40	16.52	20.62	24.70	28.76	32.80	36.82
28	*cis*-Phe-D2 → *cis*-Phe-D3	$\Delta G^{\ddagger}$	38.14	38.33	38.54	38.76	39.01	39.27	39.54	39.83	40.14	40.46	40.79	41.14	41.50
		$\Delta_r G_m^o$	31.43	31.54	31.65	31.73	31.81	31.88	31.93	31.98	32.03	32.07	32.11	32.14	32.17
29	*cis*-Phe-D3 → *cis*-Phe-D4	$\Delta G^{\ddagger}$	13.91	13.89	13.88	13.88	13.90	13.94	14.00	14.07	14.16	14.27	14.39	14.52	14.66
		$\Delta_r G_m^o$	−78.84	−79.62	−80.44	−81.27	−82.10	−82.94	−83.78	−84.62	−85.46	−86.29	−87.13	−87.96	−88.80
30	*cis*-Phe-D 4→ *cis*-Phe-D5	$\Delta G^{\ddagger}$	28.30	28.80	29.34	29.91	30.50	31.11	31.75	32.41	33.08	33.77	34.48	35.19	35.93
		$\Delta_r G_m^o$	12.37	12.92	13.47	14.02	14.57	15.11	15.65	16.19	16.72	17.25	17.78	18.31	18.84
31	*cis*-Phe-D5 → DF + $\cdot C_2H_3O_2$	$\Delta G^{\ddagger}$	10.57	10.52	10.50	10.51	10.54	10.60	10.68	10.78	10.90	11.03	11.19	11.35	11.53
		$\Delta_r G_m^o$	−27.80	−31.56	−35.58	−39.51	−43.37	−47.17	−50.92	−54.62	−58.28	−61.89	−65.47	−69.02	−72.54

Table S3. Calculated activation enthalpies and standard enthalpy changes (in kcal/mol) for the reactions from fluorene (Flu) to dibenzofuran (DF) and 9-fluorenone (9FO).

No.	Elementary Reactions		Temperature (K)												
			**298.15**	**400**	**500**	**600**	**700**	**800**	**900**	**1000**	**1100**	**1200**	**1300**	**1400**	**1500**
32	Flu + ·OH → Flu-A1	$\Delta H^{\ddagger}$	−0.28	−0.30	−0.28	−0.23	−0.17	−0.09	−0.01	0.07	0.16	0.25	0.34	0.43	0.52
		$\Delta_r H_m^o$	−18.44	−18.43	−18.33	−18.16	−17.96	−17.74	−17.49	−17.24	−16.97	−16.70	−16.43	−16.15	−15.88
33	Flu-A1 → Flu-A2	$\Delta H^{\ddagger}$	27.35	27.38	27.36	27.30	27.21	27.10	26.96	26.82	26.66	26.50	26.33	26.16	25.98
		$\Delta_r H_m^o$	−4.13	−3.93	−3.78	−3.67	−3.59	−3.54	−3.50	−3.47	−3.45	−3.44	−3.43	−3.42	−3.42
34	Flu-A2 → Flu-A3	$\Delta H^{\ddagger}$	18.33	18.01	17.75	17.53	17.35	17.20	17.07	16.96	16.85	16.74	16.64	16.54	16.43
		$\Delta_r H_m^o$	−2.51	−2.66	−2.81	−2.95	−3.06	−3.15	−3.22	−3.27	−3.30	−3.32	−3.34	−3.34	−3.34
35	Flu-A3 → Flu-A4	$\Delta H^{\ddagger}$	26.62	26.44	26.27	26.10	25.93	25.76	25.59	25.41	25.23	25.05	24.87	24.68	24.49
		$\Delta_r H_m^o$	9.24	9.17	9.15	9.16	9.17	9.19	9.21	9.23	9.25	9.26	9.27	9.29	9.30

Table S3. *Cont.*

No.	Elementary Reactions		Temperature (K)												
			**298.15**	**400**	**500**	**600**	**700**	**800**	**900**	**1000**	**1100**	**1200**	**1300**	**1400**	**1500**
36	Flu-A4 → DF + $\cdot CH_3$	$\Delta H^{\ddagger}$	18.92	18.97	18.97	18.92	18.84	18.73	18.60	18.46	18.30	18.14	17.96	17.79	17.61
		$\Delta_r H_m^o$	−11.73	−11.69	−11.74	−11.83	−11.96	−12.11	−12.28	−12.45	−12.63	−12.82	−13.01	−13.20	−13.40
37	Flu + $\cdot OCl$ → Flu-B1	$\Delta H^{\ddagger}$	4.11	4.22	4.34	4.47	4.60	4.72	4.84	4.95	5.07	5.18	5.29	5.40	5.51
		$\Delta_r H_m^o$	−2.60	−2.38	−2.10	−1.80	−1.48	−1.17	−0.85	−0.53	−0.21	0.10	0.41	0.73	1.04
38	Flu-B1 → Flu-B2	$\Delta H^{\ddagger}$	28.72	28.72	28.67	28.60	28.49	28.37	28.23	28.08	27.91	27.74	27.57	27.39	27.21
		$\Delta_r H_m^o$	0.85	1.05	1.19	1.30	1.38	1.44	1.48	1.52	1.54	1.55	1.57	1.57	1.58
39	Flu-B2 → Flu-B3	$\Delta H^{\ddagger}$	18.24	18.07	17.88	17.67	17.45	17.24	17.03	16.81	16.60	16.39	16.18	15.97	15.77
		$\Delta_r H_m^o$	−46.41	−46.58	−46.74	−46.89	−47.01	−47.11	−47.20	−47.26	−47.32	−47.37	−47.41	−47.45	−47.48
40	Flu-B3 → Flu-B4	$\Delta H^{\ddagger}$	30.01	29.85	29.69	29.54	29.38	29.22	29.06	28.88	28.71	28.54	28.36	28.17	27.99
		$\Delta_r H_m^o$	9.86	9.80	9.78	9.79	9.81	9.83	9.85	9.87	9.89	9.91	9.93	9.94	9.95
41	Flu-B4 → DF + $\cdot CClH_2$	$\Delta H^{\ddagger}$	15.35	15.32	15.25	15.15	15.02	14.88	14.72	14.55	14.38	14.20	14.02	13.83	13.64
		$\Delta_r H_m^o$	−10.71	−10.71	−10.79	−10.91	−11.06	−11.22	−11.40	−11.58	−11.77	−11.96	−12.15	−12.35	−12.55
42	Flu + $\cdot OH$ → Flu-C1 + H_2O	$\Delta H^{\ddagger}$	−1.20	−1.16	−1.06	−0.93	−0.77	−0.60	−0.42	−0.24	−0.06	0.12	0.29	0.46	0.62
		$\Delta_r H_m^o$	−35.28	−35.21	−35.18	−35.17	−35.19	−35.23	−35.30	−35.38	−35.48	−35.58	−35.69	−35.81	−35.93
43	Flu + $\cdot H$ → Flu-C1 + H_2	$\Delta H^{\ddagger}$	5.18	5.05	4.98	4.94	4.93	4.93	4.93	4.93	4.93	4.92	4.91	4.89	4.87
		$\Delta_r H_m^o$	−20.66	−20.50	−20.40	−20.35	−20.35	−20.39	−20.47	−20.58	−20.72	−20.87	−21.05	−21.23	−21.42
44	Flu + $\cdot CH_3$ → Flu-C1 + CH_4	$\Delta H^{\ddagger}$	6.19	6.04	5.99	6.00	6.04	6.11	6.19	6.27	6.36	6.45	6.53	6.61	6.68
		$\Delta_r H_m^o$	−21.77	−21.92	−22.05	−22.17	−22.27	−22.35	−22.41	−22.47	−22.52	−22.56	−22.60	−22.63	−22.65
45	Flu + O_2 → Flu-C1 + $\cdot OOH$	$\Delta H^{\ddagger}$	34.76	34.96	35.21	35.48	35.77	36.05	36.32	36.59	36.84	37.08	37.30	37.52	37.72
		$\Delta_r H_m^o$	34.14	34.23	34.31	34.38	34.42	34.43	34.42	34.40	34.35	34.29	34.23	34.15	34.07
46	Flu-C1 + O_2 → Flu-C2	$\Delta_r H_m^o$	−14.53	−14.56	−14.51	−14.41	−14.26	−14.08	−13.87	−13.65	−13.41	−13.16	−12.90	−12.64	−12.37
47	Flu-C2 → Flu-C3	$\Delta_r H_m^o$	34.63	34.50	34.38	34.26	34.14	34.00	33.86	33.71	33.55	33.39	33.22	33.05	32.88
		$\Delta_r H_m^o$	28.97	28.97	29.00	29.06	29.12	29.18	29.23	29.28	29.33	29.37	29.40	29.43	29.46
48	Flu-C3 → Flu-C4	$\Delta H^{\ddagger}$	12.87	12.81	12.71	12.59	12.45	12.30	12.13	11.96	11.79	11.61	11.43	11.24	11.05
		$\Delta_r H_m^o$	−79.56	−79.36	−79.25	−79.21	−79.20	−79.20	−79.21	−79.23	−79.24	−79.25	−79.27	−79.28	−79.29
49	Flu-C4 → Flu-C5	$\Delta H^{\ddagger}$	27.01	26.83	26.67	26.51	26.35	26.18	26.01	25.84	25.66	25.48	25.30	25.11	24.93
		$\Delta_r H_m^o$	13.91	13.86	13.87	13.89	13.92	13.95	13.97	14.00	14.02	14.04	14.06	14.07	14.08

Table S3. *Cont.*

No.	Elementary Reactions		Temperature (K)												
			298.15	400	500	600	700	800	900	1000	1100	1200	1300	1400	1500
50	Flu-C5 → DF + ·CHO	$\Delta H^{\ddagger}$	8.12	8.05	7.95	7.81	7.66	7.49	7.32	7.14	6.96	6.77	6.59	6.40	6.21
		$\Delta_r H_m^o$	−10.60	−10.69	−10.85	−11.03	−11.22	−11.42	−11.62	−11.82	−12.02	−12.23	−12.43	−12.63	−12.83
51	Flu-C2 → 9FO + ·OH	$\Delta H^{\ddagger}$	38.57	38.55	38.54	38.51	38.47	38.42	38.36	38.29	38.21	38.13	38.03	37.92	37.81
		$\Delta_r H_m^o$	−29.93	−29.74	−29.66	−29.67	−29.75	−29.89	−30.07	−30.28	−30.51	−30.76	−31.02	−31.29	−31.57
52	Flu-C1 + ·OOH → Flu-D1	$\Delta_r H_m^o$	−51.19	−51.14	−51.03	−50.85	−50.64	−50.39	−50.11	−49.81	−49.50	−49.17	−48.84	−48.49	−48.14
53	Flu-D1 → 9FO + H_2O	$\Delta H^{\ddagger}$	54.20	54.29	54.37	54.43	54.46	54.47	54.47	54.45	54.42	54.37	54.30	54.23	54.15
		$\Delta_r H_m^o$	−62.69	−62.59	−62.63	−62.77	−62.98	−63.25	−63.56	−63.89	−64.25	−64.62	−65.01	−65.40	−65.80

Table S4. Calculated Gibbs energies of activation and standard Gibbs energy changes (in kcal/mol) for the reactions from fluorene (Flu) to dibenzofuran (DF) and 9-fluorenone (9FO).

No.	Elementary Reactions		Temperature (K)												
			298.15	400	500	600	700	800	900	1000	1100	1200	1300	1400	1500
32	Flu + ·OH → Flu-A1	$\Delta G^{\ddagger}$	9.42	12.74	16.00	19.25	22.49	25.72	28.94	32.16	35.36	38.56	41.75	44.93	48.11
		$\Delta_r G_m^o$	−8.19	−4.68	−1.26	2.14	5.51	8.85	12.16	15.44	18.69	21.92	25.13	28.32	31.49
33	Flu-A1 → Flu-A2	$\Delta G^{\ddagger}$	26.97	26.84	26.71	26.58	26.47	26.37	26.28	26.22	26.16	26.13	26.10	26.09	26.09
		$\Delta_r G_m^o$	−5.61	−6.14	−6.72	−7.32	−7.93	−8.55	−9.18	−9.82	−10.45	−11.09	−11.73	−12.37	−13.00
34	Flu-A2 → Flu-A3	$\Delta G^{\ddagger}$	20.05	20.69	21.40	22.15	22.93	23.74	24.57	25.41	26.26	27.12	27.98	28.86	29.74
		$\Delta_r G_m^o$	−2.53	−2.51	−2.46	−2.38	−2.27	−2.15	−2.02	−1.89	−1.75	−1.61	−1.46	−1.32	−1.18
35	Flu-A3 → Flu-A4	$\Delta G^{\ddagger}$	28.35	28.97	29.62	30.31	31.02	31.76	32.52	33.30	34.09	34.91	35.74	36.58	37.44
		$\Delta_r G_m^o$	10.91	11.49	12.08	12.66	13.24	13.82	14.40	14.98	15.55	16.12	16.69	17.27	17.83
36	Flu-A4 → DF + $\cdot CH_3$	$\Delta G^{\ddagger}$	18.49	18.33	18.17	18.02	17.88	17.75	17.63	17.53	17.45	17.37	17.32	17.28	17.25
		$\Delta_r G_m^o$	−23.47	−26.92	−30.74	−34.53	−38.30	−42.05	−45.78	−49.50	−53.19	−56.87	−60.54	−64.18	−67.82
37	Flu + ·OCl → Flu-B1	$\Delta G^{\ddagger}$	15.15	18.90	22.56	26.19	29.80	33.39	36.97	40.54	44.09	47.63	51.16	54.69	58.20
		$\Delta_r G_m^o$	8.70	12.52	16.22	19.85	23.43	26.97	30.47	33.93	37.36	40.77	44.14	47.49	50.82
38	Flu-B1 → Flu-B2	$\Delta G^{\ddagger}$	28.59	28.55	28.51	28.49	28.48	28.48	28.51	28.55	28.60	28.67	28.75	28.85	28.96
		$\Delta_r G_m^o$	−1.21	−1.95	−2.72	−3.51	−4.32	−5.14	−5.96	−6.79	−7.62	−8.46	−9.29	−10.13	−10.96
39	Flu-B2 → Flu-B3	$\Delta G^{\ddagger}$	19.09	19.40	19.76	20.15	20.58	21.04	21.53	22.04	22.58	23.13	23.70	24.28	24.88
		$\Delta_r G_m^o$	−45.99	−45.82	−45.61	−45.37	−45.11	−44.83	−44.54	−44.24	−43.94	−43.63	−43.32	−43.00	−42.68

Table S4. *Cont.*

No.	Elementary Reactions		Temperature (K)												
			**298.15**	**400**	**500**	**600**	**700**	**800**	**900**	**1000**	**1100**	**1200**	**1300**	**1400**	**1500**
40	Flu-B3 → Flu-B4	$\Delta G^{\ddagger}$	31.93	32.62	33.33	34.07	34.84	35.63	36.44	37.27	38.11	38.98	39.85	40.74	41.65
		$\Delta_r G_m^o$	11.74	12.40	13.05	13.70	14.35	15.00	15.65	16.29	16.93	17.57	18.21	18.84	19.48
41	Flu-B4 → DF + $\cdot CClH_2$	$\Delta G^{\ddagger}$	14.79	14.60	14.43	14.27	14.14	14.02	13.92	13.84	13.78	13.73	13.70	13.68	13.68
		$\Delta_r G_m^o$	−23.74	−27.63	−31.87	−36.07	−40.25	−44.41	−48.55	−52.67	−56.77	−60.85	−64.92	−68.97	−73.01
42	Flu + ·OH → Flu-C1 + H_2O	$\Delta G^{\ddagger}$	6.46	9.07	11.62	14.14	16.64	19.12	21.57	24.01	26.42	28.82	31.21	33.58	35.94
		$\Delta_r G_m^o$	−36.19	−36.51	−36.84	−37.18	−37.51	−37.84	−38.16	−38.48	−38.78	−39.08	−39.37	−39.64	−39.92
43	Flu + ·H → Flu-C1 + H_2	$\Delta G^{\ddagger}$	11.95	14.28	16.60	18.93	21.26	23.60	25.93	28.27	30.60	32.93	35.27	37.60	39.94
		$\Delta_r G_m^o$	−21.53	−21.85	−22.20	−22.56	−22.93	−23.30	−23.66	−24.01	−24.34	−24.67	−24.98	−25.27	−25.55
44	Flu + $\cdot CH_3$ → Flu-C1 + CH_4	$\Delta G^{\ddagger}$	17.24	21.04	24.79	28.55	32.31	36.06	39.80	43.53	47.25	50.96	54.67	58.37	62.06
		$\Delta_r G_m^o$	−21.26	−21.06	−20.82	−20.57	−20.29	−20.01	−19.71	−19.40	−19.10	−18.78	−18.47	−18.15	−17.83
45	Flu + O_2 → Flu-C1 + ·OOH	$\Delta G^{\ddagger}$	43.51	46.47	49.32	52.12	54.87	57.58	60.25	62.90	65.52	68.11	70.69	73.25	75.80
		$\Delta_r G_m^o$	32.69	32.18	31.66	31.12	30.58	30.03	29.47	28.93	28.38	27.84	27.31	26.78	26.25
46	Flu-C1 + O_2 → Flu-C2	$\Delta_r G_m^o$	−3.88	−0.23	3.34	6.90	10.44	13.96	17.45	20.92	24.37	27.79	31.19	34.58	37.94
47	Flu-C2 → Flu-C3	$\Delta G^{\ddagger}$	35.82	36.24	36.69	37.16	37.66	38.17	38.70	39.24	39.81	40.38	40.97	41.57	42.19
		$\Delta_r G_m^o$	30.04	30.41	30.76	31.11	31.45	31.78	32.10	32.41	32.72	33.03	33.34	33.64	33.94
48	Flu-C3 → Flu-C4	$\Delta G^{\ddagger}$	13.21	13.34	13.48	13.64	13.83	14.04	14.27	14.51	14.78	15.06	15.35	15.66	15.98
		$\Delta_r G_m^o$	−81.82	−82.63	−83.47	−84.31	−85.17	−86.02	−86.87	−87.72	−88.57	−89.42	−90.26	−91.11	−91.95
49	Flu-C4 → Flu-C5	$\Delta G^{\ddagger}$	28.62	29.20	29.81	30.45	31.13	31.82	32.53	33.27	34.02	34.79	35.57	36.37	37.18
		$\Delta_r G_m^o$	14.99	15.37	15.75	16.13	16.50	16.87	17.23	17.59	17.95	18.31	18.66	19.01	19.37
50	Flu-C5 → DF + ·CHO	$\Delta G^{\ddagger}$	8.26	8.32	8.40	8.50	8.63	8.78	8.95	9.14	9.35	9.58	9.82	10.07	10.34
		$\Delta_r G_m^o$	−22.34	−25.78	−29.55	−33.28	−36.97	−40.63	−44.28	−47.89	−51.49	−55.07	−58.63	−62.18	−65.71
51	Flu-C2 → 9FO + ·OH	$\Delta G^{\ddagger}$	38.93	39.05	39.18	39.31	39.44	39.59	39.74	39.89	40.06	40.23	40.40	40.59	40.79
		$\Delta_r G_m^o$	−40.00	−43.48	−46.93	−50.38	−53.82	−57.25	−60.66	−64.05	−67.42	−70.76	−74.08	−77.39	−80.67
52	Flu-C1 + ·OOH → Flu-D1	$\Delta_r G_m^o$	−38.77	−34.53	−30.39	−26.28	−22.20	−18.15	−14.14	−10.16	−6.21	−2.29	1.61	5.48	9.32
53	Flu-D1 → 9FO + H_2O	$\Delta G^{\ddagger}$	53.85	53.71	53.56	53.39	53.22	53.04	52.86	52.68	52.51	52.34	52.17	52.01	51.85
		$\Delta_r G_m^o$	−74.00	−77.88	−81.70	−85.50	−89.27	−93.01	−96.71	−100.38	−104.01	−107.60	−111.17	−114.71	−118.22

Table S5. Calculated activation enthalpies and standard enthalpy changes (in kcal/mol) for the reactions from 9-methylfluorene (9MF) to dibenzofuran (DF) and 9-fluorenone (9FO).

No.	Elementary Reactions		Temperature (K)												
			**298.15**	**400**	**500**	**600**	**700**	**800**	**900**	**1000**	**1100**	**1200**	**1300**	**1400**	**1500**
54	9MF + ·OH → *trans*−9MF-A1	$\Delta H^{\ddagger}$	−0.42	−0.43	−0.39	−0.33	−0.26	−0.17	−0.09	0.00	0.09	0.19	0.28	0.37	0.47
		$\Delta_r H_m^o$	−19.10	−19.08	−18.96	−18.78	−18.57	−18.33	−18.08	−17.82	−17.55	−17.27	−17.00	−16.72	−16.43
55	*trans*−9MF-A1 → 9MF-A2	$\Delta H^{\ddagger}$	24.94	24.95	24.91	24.82	24.71	24.58	24.44	24.28	24.12	23.95	23.78	23.60	23.42
		$\Delta_r H_m^o$	−4.66	−4.50	−4.40	−4.34	−4.29	−4.27	−4.25	−4.24	−4.23	−4.23	−4.23	−4.23	−4.23
56	9MF-A2 → 9MF-A3	$\Delta H^{\ddagger}$	18.65	18.35	18.10	17.89	17.72	17.58	17.46	17.35	17.24	17.14	17.04	16.93	16.82
		$\Delta_r H_m^o$	1.09	0.92	0.76	0.63	0.52	0.44	0.38	0.34	0.31	0.29	0.28	0.28	0.28
57	9MF-A3 → 9MF-A4	$\Delta H^{\ddagger}$	25.74	25.55	25.39	25.22	25.06	24.89	24.71	24.53	24.35	24.17	23.99	23.80	23.61
		$\Delta_r H_m^o$	7.96	7.9	7.88	7.89	7.91	7.93	7.96	7.98	7.99	8.01	8.02	8.03	8.04
58	9MF-A4 → DF + $\cdot C_2H_5$	$\Delta H^{\ddagger}$	16.32	16.31	16.25	16.17	16.05	15.91	15.77	15.61	15.44	15.27	15.09	14.91	14.73
		$\Delta_r H_m^o$	−10.63	−10.66	−10.75	−10.88	−11.03	−11.2	−11.38	−11.56	−11.75	−11.95	−12.14	−12.34	−12.53
59	9MF + ·OH → *cis*−9MF-A1	$\Delta H^{\ddagger}$	0.13	0.13	0.17	0.23	0.30	0.38	0.47	0.56	0.65	0.74	0.83	0.93	1.02
		$\Delta_r H_m^o$	−18.86	−18.84	−18.72	−18.54	−18.33	−18.09	−17.84	−17.58	−17.31	−17.04	−16.76	−16.48	−16.20
60	*cis*−9MF-A1 → 9MF-A2	$\Delta H^{\ddagger}$	23.87	23.85	23.79	23.70	23.58	23.45	23.30	23.14	22.98	22.81	22.63	22.46	22.28
		$\Delta_r H_m^o$	−4.90	−4.74	−4.64	−4.58	−4.53	−4.51	−4.49	−4.48	−4.47	−4.46	−4.46	−4.46	−4.46
61	9MF + ·OCl → *trans*−9MF-B1	$\Delta H^{\ddagger}$	3.82	3.95	4.09	4.22	4.36	4.49	4.62	4.74	4.86	4.97	5.08	5.19	5.30
		$\Delta_r H_m^o$	−3.37	−3.14	−2.85	−2.53	−2.21	−1.88	−1.56	−1.23	−0.91	−0.59	−0.28	0.04	0.35
62	*trans*−9MF-B1 → 9MF-B2	$\Delta H^{\ddagger}$	26.43	26.41	26.34	26.24	26.11	25.97	25.82	25.66	25.49	25.32	25.14	24.96	24.78
		$\Delta_r H_m^o$	0.33	0.49	0.59	0.66	0.70	0.74	0.76	0.77	0.78	0.79	0.79	0.80	0.80
63	9MF-B2 → 9MF-B3	$\Delta H^{\ddagger}$	12.81	12.68	12.53	12.36	12.19	12.00	11.82	11.63	11.44	11.25	11.05	10.86	10.66
		$\Delta_r H_m^o$	−44.44	−44.56	−44.66	−44.75	−44.82	−44.89	−44.94	−44.98	−45.02	−45.05	−45.08	−45.11	−45.13
64	9MF-B3 → 9MF-B4	$\Delta H^{\ddagger}$	29.15	28.99	28.83	28.68	28.52	28.35	28.18	28.01	27.83	27.65	27.47	27.28	27.10
		$\Delta_r H_m^o$	7.83	7.76	7.75	7.75	7.77	7.79	7.81	7.83	7.85	7.87	7.88	7.89	7.90
65	9MF-B4 → DF + $\cdot C_2ClH_4$	$\Delta H^{\ddagger}$	13.29	13.23	13.12	12.98	12.83	12.66	12.48	12.30	12.12	11.93	11.75	11.56	11.37
		$\Delta_r H_m^o$	−10.45	−10.55	−10.70	−10.87	−11.05	−11.25	−11.45	−11.65	−11.85	−12.05	−12.25	−12.46	−12.66

Table S5. *Cont.*

No.	Elementary Reactions		Temperature (K)												
			**298.15**	**400**	**500**	**600**	**700**	**800**	**900**	**1000**	**1100**	**1200**	**1300**	**1400**	**1500**
66	9MF + ·OCl → *cis*−9MF-B1	$\Delta H^{\ddagger}$	4.86	4.99	5.13	5.26	5.40	5.53	5.65	5.77	5.89	6.01	6.12	6.23	6.34
		$\Delta_r H_m^o$	−2.60	−2.36	−2.08	−1.76	−1.44	−1.11	−0.79	−0.46	−0.14	0.17	0.49	0.80	1.12
67	*cis*−9MF-B1 → 9MF-B2	$\Delta H^{\ddagger}$	27.09	27.04	26.95	26.84	26.71	26.56	26.41	26.24	26.07	25.90	25.72	25.54	25.35
		$\Delta_r H_m^o$	−0.44	−0.28	−0.18	−0.11	−0.07	−0.03	−0.01	0.00	0.01	0.02	0.03	0.03	0.03
68	9MF + ·OH → 9MF-C1 + H_2O	$\Delta H^{\ddagger}$	−2.33	−2.28	−2.18	−2.05	−1.9	−1.73	−1.56	−1.38	−1.21	−1.04	−0.87	−0.71	−0.55
		$\Delta_r H_m^o$	−39.31	−39.21	−39.16	−39.15	−39.17	−39.22	−39.29	−39.37	−39.46	−39.57	−39.68	−39.79	−39.91
69	9MF + ·H → 9MF-C1 + H_2	$\Delta H^{\ddagger}$	3.44	3.34	3.28	3.25	3.24	3.24	3.24	3.24	3.24	3.23	3.22	3.20	3.18
		$\Delta_r H_m^o$	−24.69	−24.50	−24.38	−24.33	−24.33	−24.37	−24.46	−24.57	−24.71	−24.86	−25.03	−25.21	−25.41
70	9MF + ·CH_3 → 9MF-C1 + CH_4	$\Delta H^{\ddagger}$	5.14	5.25	5.42	5.64	5.90	6.17	6.46	6.75	7.04	7.33	7.61	7.89	8.17
		$\Delta_r H_m^o$	−25.80	−25.91	−26.03	−26.15	−26.25	−26.33	−26.40	−26.46	−26.51	−26.55	−26.58	−26.61	−26.63
71	9MF + O_2 → 9MF-C1 + ·OOH	$\Delta H^{\ddagger}$	32.09	32.33	32.60	32.88	33.16	33.44	33.71	33.97	34.22	34.46	34.69	34.90	35.11
		$\Delta_r H_m^o$	30.11	30.24	30.33	30.40	30.44	30.45	30.44	30.41	30.36	30.31	30.24	30.17	30.09
72	9MF-C1 + O_2 → 9MF-C2	$\Delta_r H_m^o$	−14.29	−14.29	−14.19	−14.03	−13.84	−13.62	−13.38	−13.13	−12.87	−12.61	−12.34	−12.07	−11.80
73	9MF-C2 → 9MF-C3	$\Delta_r H_m^o$	33.30	33.19	33.07	32.96	32.83	32.69	32.55	32.40	32.24	32.08	31.91	31.74	31.57
		$\Delta_r H_m^o$	27.15	27.16	27.20	27.25	27.31	27.36	27.41	27.46	27.50	27.53	27.57	27.60	27.62
74	9MF-C3 → 9MF-C4	$\Delta H^{\ddagger}$	11.94	11.85	11.72	11.58	11.42	11.25	11.07	10.89	10.71	10.53	10.34	10.15	9.96
		$\Delta_r H_m^o$	−80.21	−80.04	−79.96	−79.93	−79.92	−79.93	−79.95	−79.97	−79.99	−80.01	−80.03	−80.05	−80.07
75	9MF-C4 → 9MF-C5	$\Delta H^{\ddagger}$	27.15	26.97	26.81	26.64	26.48	26.31	26.14	25.97	25.79	25.60	25.42	25.23	25.05
		$\Delta_r H_m^o$	13.30	13.25	13.25	13.27	13.30	13.33	13.35	13.38	13.40	13.42	13.43	13.45	13.46
76	9MF-C5 → DF + ·$COCH_3$	$\Delta H^{\ddagger}$	7.81	7.73	7.60	7.44	7.27	7.08	6.89	6.69	6.49	6.29	6.09	5.89	5.69
		$\Delta_r H_m^o$	−10.27	−10.45	−10.67	−10.90	−11.13	−11.36	−11.59	−11.81	−12.03	−12.25	−12.47	−12.69	−12.90
77	9MF-C1 + ·OOH → 9MF-D1	$\Delta_r H_m^o$	−50.60	−50.53	−50.37	−50.14	−49.88	−49.59	−49.28	−48.96	−48.63	−48.29	−47.94	−47.59	−47.23
78	9MF-D1 → 9MF-D2 + ·OH	$\Delta_r H_m^o$	44.34	44.50	44.57	44.57	44.52	44.41	44.28	44.11	43.93	43.73	43.52	43.30	43.06
79	9MF-D2 → 9FO + ·CH_3	$\Delta H^{\ddagger}$	11.34	11.40	11.41	11.38	11.32	11.23	11.11	10.98	10.84	10.68	10.51	10.34	10.17
		$\Delta_r H_m^o$	3.60	3.74	3.75	3.65	3.48	3.25	2.98	2.68	2.36	2.03	1.68	1.32	0.95

Table S6. Calculated Gibbs energies of activation and standard Gibbs energy changes (in kcal/mol) for the reactions from 9-methylfluorene (9MF) to dibenzofuran (DF) and 9-fluorenone (9FO).

No.	Elementary Reactions		Temperature (K)												
			**298.15**	**400**	**500**	**600**	**700**	**800**	**900**	**1000**	**1100**	**1200**	**1300**	**1400**	**1500**
54	9MF + ·OH → *trans*−9MF-A1	$\Delta G^{\ddagger}$	9.28	12.60	15.85	19.09	22.32	25.54	28.75	31.95	35.14	38.33	41.50	44.67	47.83
		$\Delta_r G_m^o$	−8.72	−5.17	−1.71	1.73	5.13	8.50	11.84	15.14	18.43	21.69	24.92	28.14	31.33
55	*trans*−9MF-A1 → 9MF-A2	$\Delta G^{\ddagger}$	24.08	23.78	23.49	23.21	22.95	22.71	22.48	22.27	22.08	21.90	21.74	21.59	21.45
		$\Delta_r G_m^o$	−6.85	−7.63	−8.42	−9.23	−10.05	−10.88	−11.71	−12.54	−13.36	−14.20	−15.03	−15.86	−16.69
56	9MF-A2 → 9MF-A3	$\Delta G^{\ddagger}$	20.96	21.80	22.69	23.63	24.60	25.60	26.61	27.63	28.66	29.70	30.76	31.81	32.88
		$\Delta_r G_m^o$	1.46	1.61	1.80	2.02	2.26	2.52	2.78	3.05	3.32	3.60	3.87	4.15	4.43
57	9MF-A3 → 9MF-A4	$\Delta G^{\ddagger}$	27.74	28.45	29.19	29.97	30.78	31.60	32.45	33.32	34.21	35.12	36.04	36.97	37.92
		$\Delta_r G_m^o$	9.78	10.41	11.04	11.67	12.30	12.92	13.55	14.17	14.79	15.40	16.02	16.63	17.25
58	9MF-A4 → DF + $\cdot C_2H_5$	$\Delta G^{\ddagger}$	15.80	15.63	15.46	15.31	15.18	15.07	14.97	14.89	14.83	14.78	14.74	14.72	14.72
		$\Delta_r G_m^o$	−23.43	−27.23	−31.38	−35.50	−39.59	−43.66	−47.70	−51.73	−55.74	−59.73	−63.70	−67.66	−71.61
59	9MF + ·OH → *cis*−9MF-A1	$\Delta G^{\ddagger}$	9.77	13.06	16.29	19.51	22.72	25.91	29.10	32.28	35.44	38.60	41.75	44.90	48.04
		$\Delta_r G_m^o$	−8.51	−4.97	−1.51	1.91	5.30	8.66	11.99	15.29	18.57	21.82	25.04	28.25	31.43
60	*cis*−9MF-A1 → 9MF-A2	$\Delta G^{\ddagger}$	23.13	22.87	22.64	22.41	22.21	22.02	21.85	21.70	21.56	21.44	21.33	21.24	21.16
		$\Delta_r G_m^o$	−7.07	−7.83	−8.62	−9.42	−10.23	−11.04	−11.86	−12.68	−13.50	−14.33	−15.15	−15.97	−16.79
61	9MF + ·OCl → *trans*−9MF-B1	$\Delta G^{\ddagger}$	14.91	18.68	22.34	25.98	29.59	33.19	36.77	40.34	43.89	47.43	50.97	54.49	58.01
		$\Delta_r G_m^o$	8.06	11.93	15.66	19.34	22.96	26.53	30.06	33.56	37.02	40.45	43.86	47.25	50.61
62	*trans*−9MF-B1 → 9MF-B2	$\Delta G^{\ddagger}$	26.15	26.06	25.98	25.92	25.87	25.85	25.84	25.85	25.88	25.92	25.98	26.05	26.14
		$\Delta_r G_m^o$	−2.45	−3.42	−4.41	−5.42	−6.44	−7.46	−8.49	−9.51	−10.54	−11.57	−12.60	−13.63	−14.66
63	9MF-B2 → 9MF-B3	$\Delta G^{\ddagger}$	13.19	13.34	13.52	13.73	13.97	14.24	14.53	14.84	15.17	15.52	15.89	16.26	16.66
		$\Delta_r G_m^o$	−43.72	−43.46	−43.17	−42.86	−42.54	−42.21	−41.87	−41.53	−41.18	−40.83	−40.48	−40.12	−39.76
64	9MF-B3 → 9MF-B4	$\Delta G^{\ddagger}$	31.57	32.42	33.29	34.20	35.13	36.09	37.07	38.06	39.08	40.11	41.15	42.21	43.29
		$\Delta_r G_m^o$	10.25	11.09	11.93	12.76	13.60	14.43	15.26	16.08	16.91	17.73	18.55	19.37	20.19
65	9MF-B4 → DF + $\cdot C_2ClH_4$	$\Delta G^{\ddagger}$	12.72	12.54	12.38	12.24	12.13	12.05	11.98	11.93	11.91	11.89	11.90	11.92	11.95
		$\Delta_r G_m^o$	−23.72	−27.68	−31.95	−36.19	−40.40	−44.57	−48.73	−52.86	−56.97	−61.07	−65.14	−69.20	−73.25
66	9MF + ·OCl → *cis*−9MF-B1	$\Delta G^{\ddagger}$	15.95	19.73	23.39	27.03	30.65	34.25	37.83	41.4	44.96	48.51	52.04	55.57	59.09
		$\Delta_r G_m^o$	8.82	12.69	16.42	20.09	23.70	27.27	30.80	34.29	37.75	41.18	44.59	47.97	51.33

Table S6. *Cont.*

No.	Elementary Reactions		Temperature (K)												
			**298.15**	**400**	**500**	**600**	**700**	**800**	**900**	**1000**	**1100**	**1200**	**1300**	**1400**	**1500**
67	*cis*−9MF-B1 → 9MF-B2	$\Delta G^{\ddagger}$	26.79	26.69	26.61	26.55	26.52	26.50	26.50	26.52	26.56	26.61	26.67	26.75	26.85
		$\Delta_r G_m^o$	−3.21	−4.18	−5.17	−6.17	−7.18	−8.20	−9.22	−10.25	−11.27	−12.30	−13.33	−14.36	−15.38
68	9MF + ·OH → 9MF-C1 + H_2O	$\Delta G^{\ddagger}$	5.88	8.67	11.40	14.11	16.78	19.44	22.08	24.69	27.29	29.88	32.45	35.01	37.55
		$\Delta_r G_m^o$	−41.84	−42.73	−43.61	−44.51	−45.40	−46.29	−47.17	−48.04	−48.90	−49.75	−50.60	−51.43	−52.26
69	9MF + ·H → 9MF-C1 + H_2	$\Delta G^{\ddagger}$	9.99	12.25	14.48	16.72	18.97	21.22	23.46	25.71	27.96	30.20	32.45	34.70	36.95
		$\Delta_r G_m^o$	−27.18	−28.06	−28.97	−29.89	−30.82	−31.74	−32.66	−33.57	−34.46	−35.34	−36.20	−37.06	−37.90
70	9MF + ·CH_3 → 9MF-C1 + CH_4	$\Delta G^{\ddagger}$	14.20	17.27	20.26	23.21	26.12	28.99	31.82	34.63	37.40	40.15	42.87	45.57	48.25
		$\Delta_r G_m^o$	−26.91	−27.27	−27.60	−27.90	−28.18	−28.45	−28.71	−28.96	−29.21	−29.46	−29.70	−29.94	−30.17
71	9MF + O_2 → 9MF-C1 + ·OOH	$\Delta G^{\ddagger}$	40.87	43.83	46.67	49.46	52.20	54.90	57.57	60.21	62.82	65.41	67.98	70.53	73.07
		$\Delta_r G_m^o$	27.04	25.97	24.89	23.79	22.69	21.58	20.47	19.37	18.27	17.17	16.08	14.99	13.91
72	9MF-C1 + O_2 → 9MF-C2	$\Delta_r G_m^o$	−1.52	2.85	7.12	11.37	15.59	19.78	23.94	28.07	32.18	36.26	40.32	44.37	48.39
73	9MF-C2 → 9MF-C3	$\Delta G^{\ddagger}$	34.09	34.38	34.69	35.03	35.38	35.75	36.15	36.55	36.98	37.41	37.87	38.33	38.81
		$\Delta_r G_m^o$	27.78	27.99	28.19	28.39	28.57	28.75	28.92	29.09	29.25	29.41	29.56	29.71	29.86
74	9MF-C3 → 9MF-C4	$\Delta G^{\ddagger}$	11.86	11.85	11.86	11.91	11.97	12.06	12.18	12.31	12.46	12.63	12.81	13.01	13.22
		$\Delta_r G_m^o$	−82.69	−83.57	−84.46	−85.36	−86.27	−87.17	−88.08	−88.98	−89.88	−90.77	−91.67	−92.57	−93.46
75	9MF-C4 → 9MF-C5	$\Delta G^{\ddagger}$	28.93	29.56	30.23	30.93	31.66	32.41	33.18	33.97	34.78	35.61	36.45	37.30	38.17
		$\Delta_r G_m^o$	14.38	14.75	15.13	15.50	15.87	16.24	16.60	16.96	17.32	17.67	18.02	18.38	18.73
76	9MF-C5 → DF + ·$COCH_3$	$\Delta G^{\ddagger}$	7.21	7.01	6.84	6.71	6.60	6.51	6.46	6.42	6.40	6.40	6.42	6.45	6.50
		$\Delta_r G_m^o$	−22.30	−25.82	−29.65	−33.43	−37.16	−40.87	−44.54	−48.19	−51.82	−55.42	−59.01	−62.58	−66.14
77	9MF-C1 + ·OOH → 9MF-D1	$\Delta_r G_m^o$	−36.01	−31.03	−26.17	−21.36	−16.58	−11.84	−7.14	−2.48	2.16	6.76	11.33	15.88	20.40
78	9MF-D1 → 9MF-D2 + ·OH	$\Delta_r G_m^o$	32.72	28.73	24.78	20.82	16.86	12.92	8.99	5.08	1.18	−2.70	−6.56	−10.40	−14.23
79	9MF-D2 → 9FO + ·CH_3	$\Delta G^{\ddagger}$	11.21	11.16	11.10	11.04	10.99	10.94	10.91	10.90	10.90	10.91	10.94	10.98	11.03
		$\Delta_r G_m^o$	−8.46	−12.61	−16.70	−20.78	−24.84	−28.87	−32.87	−36.84	−40.78	−44.68	−48.56	−52.41	−56.24

Table S7. Calculated activation enthalpies and standard enthalpy changes (in kcal/mol) for the reactions from 9-fluorenone (9FO) to dibenzofuran (DF).

No.	Elementary Reactions		Temperature (K)												
			298.15	400	500	600	700	800	900	1000	1100	1200	1300	1400	1500
80	9FO + ·OH → 9FO-A1	$\Delta H^{\ddagger}$	7.86	7.80	7.81	7.85	7.91	7.98	8.07	8.16	8.25	8.35	8.44	8.54	8.64
		$\Delta_r H_m^o$	−14.52	−14.54	−14.45	−14.30	−14.11	−13.88	−13.64	−13.38	−13.12	−12.84	−12.57	−12.29	−12.01
81	9FO-A1 → 9FO-A2	$\Delta H^{\ddagger}$	10.31	10.22	10.09	9.94	9.77	9.59	9.41	9.23	9.04	8.85	8.66	8.47	8.27
		$\Delta_r H_m^o$	−1.07	−1.06	−1.08	−1.12	−1.15	−1.19	−1.22	−1.25	−1.28	−1.31	−1.33	−1.35	−1.36
82	9FO-A2 + O^{3P} → 9FO-A3	$\Delta_r H_m^o$	−127.05	−127.16	−127.22	−127.24	−127.24	−127.22	−127.18	−127.14	−127.08	−127.02	−126.96	−126.89	−126.81
83	9FO-A3 → 9FO-A4	$\Delta H^{\ddagger}$	28.46	28.29	28.13	27.97	27.80	27.64	27.47	27.29	27.11	26.93	26.75	26.56	26.38
		$\Delta_r H_m^o$	15.45	15.40	15.39	15.40	15.42	15.44	15.47	15.49	15.51	15.52	15.54	15.55	15.56
84	9FO-A4 → DF + ·COOH	$\Delta H^{\ddagger}$	12.95	12.89	12.78	12.63	12.47	12.29	12.10	11.91	11.72	11.53	11.33	11.14	10.95
		$\Delta_r H_m^o$	4.55	4.29	3.94	3.55	3.15	2.74	2.32	1.91	1.49	1.07	0.66	0.25	−0.16
85	9FO + ·OH → 9FO-B1	$\Delta H^{\ddagger}$	0.97	0.97	1.00	1.06	1.13	1.21	1.30	1.39	1.48	1.57	1.66	1.76	1.85
		$\Delta_r H_m^o$	−16.74	−16.71	−16.59	−16.41	−16.20	−15.96	−15.71	−15.44	−15.17	−14.89	−14.61	−14.33	−14.05
86	9FO-B1 → 9FO-B2	$\Delta H^{\ddagger}$	16.84	16.77	16.67	16.53	16.38	16.21	16.03	15.85	15.66	15.47	15.28	15.09	14.90
		$\Delta_r H_m^o$	−2.70	−2.61	−2.58	−2.58	−2.60	−2.62	−2.64	−2.67	−2.69	−2.71	−2.73	−2.75	−2.77
87	9FO-B2 → Flu-C4	$\Delta H^{\ddagger}$	14.79	14.54	14.34	14.18	14.05	13.94	13.84	13.76	13.67	13.58	13.50	13.40	13.31
		$\Delta_r H_m^o$	−1.22	−1.33	−1.42	−1.48	−1.53	−1.55	−1.56	−1.56	−1.54	−1.53	−1.50	−1.48	−1.45
88	9FO-B2 → Phe-B5	$\Delta H^{\ddagger}$	24.24	24.27	24.25	24.19	24.09	23.97	23.84	23.69	23.53	23.37	23.20	23.02	22.85
		$\Delta_r H_m^o$	23.24	23.20	23.10	22.94	22.74	22.52	22.29	22.03	21.77	21.50	21.23	20.95	20.67
89	9FO + ·OCl → 9FO-C1	$\Delta H^{\ddagger}$	6.62	6.74	6.87	7.01	7.14	7.27	7.39	7.51	7.63	7.75	7.86	7.97	8.08
		$\Delta_r H_m^o$	−0.29	−0.05	0.24	0.55	0.88	1.21	1.53	1.86	2.18	2.51	2.82	3.14	3.46
90	9FO-C1 → 9FO-C2	$\Delta H^{\ddagger}$	19.13	19.07	18.96	18.83	18.67	18.50	18.32	18.14	17.95	17.76	17.57	17.38	17.18
		$\Delta_r H_m^o$	1.53	1.61	1.65	1.65	1.64	1.62	1.60	1.58	1.55	1.54	1.52	1.50	1.49
91	9FO-C2 → 9FO-C3	$\Delta H^{\ddagger}$	10.46	10.37	10.25	10.11	9.97	9.81	9.65	9.48	9.30	9.13	8.95	8.76	8.58
		$\Delta_r H_m^o$	−53.76	−53.80	−53.83	−53.84	−53.85	−53.86	−53.86	−53.86	−53.86	−53.86	−53.86	−53.86	−53.86
92	9FO-C3 → 9FO-C4	$\Delta H^{\ddagger}$	27.01	26.83	26.66	26.50	26.33	26.16	25.99	25.81	25.63	25.45	25.26	25.08	24.89
		$\Delta_r H_m^o$	12.27	12.21	12.19	12.20	12.21	12.23	12.25	12.27	12.28	12.30	12.31	12.32	12.33
93	9FO-C4 → DF + ·CClO	$\Delta H^{\ddagger}$	6.99	6.92	6.80	6.65	6.48	6.29	6.10	5.90	5.71	5.51	5.31	5.10	4.90
		$\Delta_r H_m^o$	−1.48	−1.73	−2.06	−2.44	−2.85	−3.26	−3.68	−4.10	−4.52	−4.94	−5.36	−5.77	−6.19

Table S7. *Cont.*

No.	Elementary Reactions		Temperature (K)												
			**298.15**	**400**	**500**	**600**	**700**	**800**	**900**	**1000**	**1100**	**1200**	**1300**	**1400**	**1500**
94	9FO-C2 → Phe-C5	$\Delta H^{\ddagger}$	24.11	24.15	24.12	24.06	23.97	23.85	23.71	23.57	23.41	23.25	23.08	22.91	22.73
		$\Delta_r H_m^o$	23.60	23.56	23.45	23.30	23.10	22.89	22.65	22.40	22.14	21.87	21.60	21.32	21.04
95	9FO + ·OH → 9FO-D1	$\Delta H^{\ddagger}$	18.09	18.19	18.31	18.44	18.57	18.71	18.84	18.97	19.10	19.22	19.35	19.47	19.58
		$\Delta_r H_m^o$	3.15	3.32	3.56	3.84	4.14	4.45	4.77	5.08	5.40	5.71	6.03	6.34	6.65
96	9FO-D1 → 9FO-D2	$\Delta H^{\ddagger}$	11.77	11.72	11.62	11.49	11.34	11.18	11.00	10.83	10.64	10.46	10.27	10.08	9.89
		$\Delta_r H_m^o$	2.54	2.60	2.62	2.61	2.60	2.58	2.56	2.54	2.52	2.50	2.49	2.47	2.46
97	9FO-D2 + O^{3P} → 9FO-D3	$\Delta_r H_m^o$	−126.66	−126.77	−126.83	−126.85	−126.85	−126.83	−126.79	−126.75	−126.70	−126.63	−126.57	−126.50	−126.42
98	9FO-D3 → DF + ·Cl + CO_2	$\Delta H^{\ddagger}$	29.64	29.46	29.30	29.14	28.98	28.81	28.63	28.46	28.28	28.10	27.91	27.73	27.54
		$\Delta_r H_m^o$	−61.43	−61.75	−62.13	−62.54	−62.98	−63.42	−63.87	−64.32	−64.76	−65.21	−65.65	−66.09	−66.53
99	9FO-A2 + O_2 → 9FO-S1	$\Delta_r H_m^o$	−46.54	−46.50	−46.39	−46.22	−46.02	−45.80	−45.56	−45.30	−45.04	−44.78	−44.51	−44.23	−43.95
100	9FO-S1 → 9FO-S2	$\Delta H^{\ddagger}$	30.49	30.34	30.21	30.10	30.00	29.91	29.83	29.75	29.66	29.58	29.49	29.40	29.30
		$\Delta_r H_m^o$	23.18	23.28	23.37	23.44	23.50	23.55	23.60	23.64	23.67	23.70	23.73	23.75	23.78
101	9FO-S2 → 9FO-S3	$\Delta H^{\ddagger}$	6.32	6.28	6.20	6.08	5.95	5.79	5.63	5.45	5.27	5.09	4.90	4.71	4.52
		$\Delta_r H_m^o$	−2.54	−2.67	−2.85	−3.08	−3.33	−3.61	−3.90	−4.20	−4.50	−4.81	−5.12	−5.43	−5.74
102	9FO-S3 → DF + ·OH	$\Delta H^{\ddagger}$	13.48	13.47	13.42	13.34	13.24	13.12	12.98	12.83	12.67	12.51	12.34	12.17	11.99
		$\Delta_r H_m^o$	−62.34	−62.46	−62.64	−62.84	−63.06	−63.31	−63.56	−63.83	−64.10	−64.38	−64.66	−64.94	−65.23
103	9FO-A2 → 9FO-S4	$\Delta H^{\ddagger}$	11.63	11.49	11.38	11.28	11.20	11.12	11.06	10.99	10.92	10.85	10.77	10.69	10.60
		$\Delta_r H_m^o$	−0.95	−0.99	−1.03	−1.06	−1.07	−1.08	−1.08	−1.08	−1.07	−1.06	−1.04	−1.02	−1.01
104	9FO-S4 → 9FO-S5 + CO_2	$\Delta H^{\ddagger}$	5.84	5.80	5.73	5.62	5.49	5.34	5.18	5.01	4.83	4.65	4.46	4.28	4.09
		$\Delta_r H_m^o$	−5.77	−5.91	−6.09	−6.32	−6.58	−6.86	−7.15	−7.45	−7.76	−8.06	−8.37	−8.68	−8.99
105	9FO-S5 + O_2 → Phe-S6	$\Delta_r H_m^o$	−44.81	−44.77	−44.66	−44.49	−44.29	−44.06	−43.82	−43.56	−43.30	−43.04	−42.76	−42.49	−42.21
106	9FO-S6 → 9FO-S3	$\Delta H^{\ddagger}$	28.32	28.28	28.26	28.25	28.23	28.22	28.19	28.15	28.10	28.04	27.97	27.89	27.80
		$\Delta_r H_m^o$	−2.68	−2.50	−2.36	−2.24	−2.15	−2.07	−1.99	−1.92	−1.85	−1.77	−1.69	−1.60	−1.51
107	9FO-S5 + O^{3P} → 9FO-S7	$\Delta_r H_m^o$	−125.14	−125.26	−125.32	−125.34	−125.34	−125.32	−125.29	−125.24	−125.19	−125.13	−125.06	−125.00	−124.92
108	9FO-S7 → 9FO-S8	$\Delta H^{\ddagger}$	26.77	26.56	26.38	26.19	26.01	25.83	25.64	25.46	25.27	25.09	24.90	24.72	24.53

Table S7. *Cont.*

No.	Elementary Reactions		Temperature (K)												
			**298.15**	**298.15**	**298.15**	**298.15**	**298.15**	**298.15**	**298.15**	**298.15**	**298.15**	**298.15**	**298.15**	**298.15**	**298.15**
		$\Delta_r H_m^o$	12.37	12.26	12.19	12.16	12.13	12.12	12.12	12.13	12.13	12.14	12.15	12.16	12.17
109	9FO-S8 → DF + ·H	$\Delta H^{\ddagger}$	20.91	20.95	20.98	20.99	20.99	20.98	20.94	20.89	20.83	20.75	20.67	20.57	20.46
		$\Delta_r H_m^o$	13.78	14.07	14.28	14.45	14.59	14.70	14.79	14.85	14.90	14.94	14.96	14.96	14.96
110	9FO-D2 + O_2 → 9FO-S9	$\Delta_r H_m^o$	−45.68	−45.65	−45.54	−45.37	−45.17	−44.94	−44.70	−44.45	−44.19	−43.92	−43.65	−43.38	−43.10
111	9FO-S9 → 9FO-S10 + CO_2	$\Delta H^{\ddagger}$	35.50	35.40	35.29	35.17	35.04	34.90	34.75	34.60	34.44	34.27	34.10	33.93	33.75
		$\Delta_r H_m^o$	17.39	−17.93	−17.75	−17.56	−17.37	−17.18	−16.98	−16.78	−16.59	−16.39	−16.19	−15.99	−15.80
112	9FO-S10 → 9FO-S11	$\Delta H^{\ddagger}$	7.26	7.22	7.15	7.05	6.92	6.78	6.63	6.46	6.30	6.12	5.94	5.76	5.58
		$\Delta_r H_m^o$	−4.51	−4.85	−5.24	−5.67	−6.13	−6.60	−7.09	−7.59	−8.09	−8.60	−9.11	−9.61	−10.12
113	9FO-S11 → DF + ClO·	$\Delta H^{\ddagger}$	16.26	16.43	16.54	16.62	16.68	16.73	16.76	16.79	16.81	16.83	16.85	16.86	16.87
		$\Delta_r H_m^o$	−92.97	−93.26	−93.53	−93.77	−93.98	−94.17	−94.35	−94.51	−94.67	−94.82	−94.96	−95.10	−95.23
114	9FO-D2 → 9FO-S12	$\Delta H^{\ddagger}$	21.83	21.71	21.57	21.42	21.26	21.10	20.93	20.76	20.58	20.40	20.22	20.03	19.85
		$\Delta_r H_m^o$	−46.63	−46.61	−46.59	−46.58	−46.57	−46.56	−46.56	−46.55	−46.55	−46.55	−46.54	−46.54	−46.54
115	9FO-S12 → 9FO-S13 + CO_2	$\Delta H^{\ddagger}$	6.78	6.74	6.65	6.54	6.41	6.27	6.11	5.94	5.77	5.59	5.41	5.23	5.04
		$\Delta_r H_m^o$	−3.83	−3.98	−4.18	−4.42	−4.69	−4.98	−5.27	−5.58	−5.89	−6.20	−6.51	−6.82	−7.13
116	9FO-S13 + O_2 → Phe-S14	$\Delta_r H_m^o$	−45.71	−45.66	−45.54	−45.37	−45.17	−44.94	−44.69	−44.44	−44.17	−43.91	−43.63	−43.36	−43.08
117	9FO-S14 → 9FO-S15	$\Delta H^{\ddagger}$	30.51	30.46	30.43	30.42	30.40	30.38	30.35	30.31	30.26	30.20	30.13	30.05	29.96
		$\Delta_r H_m^o$	26.31	26.45	26.58	26.68	26.76	26.82	26.86	26.90	26.92	26.94	26.95	26.96	26.96
118	9FO-S15 → 1CDF + ·OH	$\Delta H^{\ddagger}$	13.43	13.40	13.34	13.25	13.14	13.02	12.87	12.72	12.56	12.39	12.22	12.04	11.86
		$\Delta_r H_m^o$	−64.71	−64.84	−65.01	−65.22	−65.45	−65.70	−65.96	−66.23	−66.51	−66.79	−67.07	−67.35	−67.64
119	9FO-S13 + O^{3P} → 9FO-S16	$\Delta_r H_m^o$	−125.40	−125.50	−125.56	−125.58	−125.57	−125.55	−125.51	−125.47	−125.41	−125.35	−125.29	−125.22	−125.14
120	9FO-S16 → 9FO-S17	$\Delta H^{\ddagger}$	25.75	25.53	25.33	25.14	24.95	24.76	24.57	24.38	24.19	24.00	23.81	23.62	23.43
		$\Delta_r H_m^o$	10.14	10.02	9.95	9.90	9.87	9.85	9.84	9.84	9.84	9.85	9.85	9.86	9.87
121	9FO-S17 → 1CDF + ·H	$\Delta H^{\ddagger}$	21.03	21.07	21.11	21.12	21.12	21.11	21.07	21.03	20.96	20.89	20.80	20.71	20.60
		$\Delta_r H_m^o$	13.67	13.96	14.18	14.35	14.49	14.60	14.69	14.76	14.81	14.84	14.86	14.87	14.87
122	9FO-S16 → DF + ·Cl	$\Delta H^{\ddagger}$	20.40	20.35	20.26	20.15	20.01	19.86	19.69	19.52	19.34	19.15	18.96	18.77	18.57
		$\Delta_r H_m^o$	−22.37	−22.45	−22.58	−22.72	−22.86	−23.01	−23.16	−23.31	−23.45	−23.59	−23.73	−23.87	−24.00

Table S8. Calculated Gibbs energies of activation and standard Gibbs energy changes (in kcal/mol) for the reactions from 9-fluorenone (9FO) to dibenzofuran (DF).

No.	Elementary Reactions		Temperature (K)												
			298.15	400	500	600	700	800	900	1000	1100	1200	1300	1400	1500
80	9FO + ·OH → 9FO-A1	$\Delta G^{\ddagger}$	17.96	21.42	24.82	28.22	31.61	35.00	38.37	41.73	45.08	48.43	51.76	55.09	58.41
		$\Delta_r G_m^o$	−4.37	−0.89	2.51	5.89	9.24	12.56	15.85	19.11	22.35	25.56	28.75	31.91	35.06
81	9FO-A1 → 9FO-A2	$\Delta G^{\ddagger}$	10.27	10.27	10.30	10.36	10.44	10.55	10.68	10.84	11.00	11.19	11.39	11.61	11.84
		$\Delta_r G_m^o$	−2.75	−3.33	−3.89	−4.45	−5.00	−5.55	−6.09	−6.63	−7.17	−7.71	−8.24	−8.77	−9.30
82	9FO-A2 + O^{3P} → 9FO-A3	$\Delta_r G_m^o$	−116.86	−113.37	−109.91	−106.45	−102.98	−99.52	−96.06	−92.60	−89.15	−85.70	−82.26	−78.83	−75.40
83	9FO-A3 → 9FO-A4	$\Delta G^{\ddagger}$	29.91	30.44	30.99	31.58	32.20	32.83	33.50	34.17	34.87	35.58	36.31	37.06	37.81
		$\Delta_r G_m^o$	16.02	16.23	16.44	16.64	16.85	17.05	17.25	17.45	17.64	17.84	18.03	18.22	18.41
84	9FO-A4 → DF + ·COOH	$\Delta G^{\ddagger}$	12.67	12.58	12.52	12.48	12.47	12.48	12.51	12.57	12.64	12.74	12.84	12.97	13.11
		$\Delta_r G_m^o$	−7.39	−10.87	−14.63	−18.31	−21.93	−25.48	−28.98	−32.44	−35.85	−39.23	−42.57	−45.88	−49.16
85	9FO + ·OH → 9FO-B1	$\Delta G^{\ddagger}$	10.36	13.56	16.71	19.84	22.97	26.08	29.19	32.28	35.36	38.44	41.51	44.57	47.63
		$\Delta_r G_m^o$	−6.69	−3.25	0.10	3.42	6.71	9.97	13.19	16.39	19.56	22.70	25.82	28.92	32.00
86	9FO-B1 → 9FO-B2	$\Delta G^{\ddagger}$	16.86	16.87	16.91	16.97	17.05	17.16	17.29	17.44	17.61	17.79	17.99	18.21	18.44
		$\Delta_r G_m^o$	−4.43	−5.04	−5.65	−6.26	−6.87	−7.48	−8.09	−8.70	−9.30	−9.90	−10.49	−11.09	−11.69
87	9FO-B2 → Flu-C4	$\Delta G^{\ddagger}$	16.72	17.42	18.16	18.94	19.75	20.57	21.40	22.25	23.10	23.96	24.83	25.70	26.59
		$\Delta_r G_m^o$	−0.66	−0.46	−0.23	0.02	0.27	0.53	0.79	1.05	1.31	1.57	1.83	2.08	2.34
88	9FO-B2 → Phe-B5	$\Delta G^{\ddagger}$	22.53	21.94	21.36	20.79	20.23	19.69	19.16	18.65	18.15	17.67	17.20	16.74	16.30
		$\Delta_r G_m^o$	12.33	8.60	4.97	1.36	−2.23	−5.78	−9.30	−12.80	−16.27	−19.72	−23.14	−26.54	−29.93
89	9FO + ·OCl → 9FO-C1	$\Delta G^{\ddagger}$	17.28	20.91	24.44	27.94	31.41	34.87	38.32	41.75	45.16	48.57	51.97	55.36	58.74
		$\Delta_r G_m^o$	10.87	14.65	18.29	21.88	25.40	28.89	32.33	35.73	39.10	42.44	45.76	49.05	52.32
90	9FO-C1 → 9FO-C2	$\Delta G^{\ddagger}$	18.79	18.68	18.60	18.54	18.50	18.49	18.50	18.52	18.57	18.64	18.72	18.81	18.92
		$\Delta_r G_m^o$	−0.64	−1.40	−2.16	−2.92	−3.68	−4.44	−5.19	−5.95	−6.70	−7.45	−8.19	−8.94	−9.69
91	9FO-C2 → 9FO-C3	$\Delta G^{\ddagger}$	11.33	11.63	11.96	12.32	12.70	13.10	13.52	13.96	14.42	14.89	15.38	15.88	16.39
		$\Delta_r G_m^o$	−53.11	−52.88	−52.65	−52.41	−52.17	−51.93	−51.69	−51.45	−51.21	−50.97	−50.73	−50.48	−50.24
92	9FO-C3 → 9FO-C4	$\Delta G^{\ddagger}$	28.96	29.66	30.38	31.14	31.93	32.74	33.58	34.43	35.30	36.19	37.09	38.01	38.94
		$\Delta_r G_m^o$	13.63	14.10	14.58	15.05	15.53	16.00	16.47	16.94	17.40	17.87	18.33	18.79	19.26
93	9FO-C4 → DF + ·CClO	$\Delta G^{\ddagger}$	6.63	6.52	6.43	6.37	6.34	6.33	6.35	6.38	6.44	6.52	6.61	6.72	6.84
		$\Delta_r G_m^o$	−13.91	−17.55	−21.49	−25.33	−29.12	−32.84	−36.52	−40.14	−43.72	−47.27	−50.78	−54.26	−57.71

Table S8. *Cont.*

No.	Elementary Reactions		Temperature (K)												
			**298.15**	**400**	**500**	**600**	**700**	**800**	**900**	**1000**	**1100**	**1200**	**1300**	**1400**	**1500**
94	9FO-C2 → Phe-C5	$\Delta G^\ddagger$	22.28	21.64	21.02	20.40	19.80	19.21	18.64	18.08	17.54	17.02	16.50	16.00	15.52
		$\Delta_r G_m^o$	12.57	8.80	5.13	1.47	−2.15	−5.74	−9.31	−12.84	−16.35	−19.84	−23.31	−26.75	−30.17
95	9FO + ·OH → 9FO-D1	$\Delta G^\ddagger$	29.26	33.07	36.77	40.46	44.11	47.76	51.38	54.98	58.58	62.16	65.74	69.30	72.86
		$\Delta_r G_m^o$	14.40	18.21	21.91	25.55	29.15	32.70	36.21	39.69	43.13	46.55	49.94	53.31	56.65
96	9FO-D1 → 9FO-D2	$\Delta G^\ddagger$	11.44	11.33	11.24	11.18	11.14	11.12	11.12	11.15	11.19	11.25	11.32	11.41	11.51
		$\Delta_r G_m^o$	0.47	−0.25	−0.96	−1.68	−2.39	−3.10	−3.81	−4.52	−5.23	−5.93	−6.63	−7.33	−8.03
97	9FO-D2 + O^{3P} → 9FO-D3	$\Delta_r G_m^o$	−116.45	−112.94	−109.48	−106.00	−102.53	−99.06	−95.59	−92.12	−88.66	−85.21	−81.76	−78.31	−74.87
98	9FO-D 3→ DF + ·Cl + CO_2	$\Delta G^\ddagger$	30.97	31.46	31.97	32.52	33.10	33.70	34.32	34.96	35.62	36.30	36.99	37.69	38.41
		$\Delta_r G_m^o$	−79.49	−85.05	−90.85	−96.56	−102.19	−107.76	−113.28	−118.74	−124.16	−129.55	−134.89	−140.20	−145.48
99	9FO-A2 + O_2 → 9FO-S1	$\Delta_r G_m^o$	−34.96	−31.01	−27.15	−23.31	−19.51	−15.74	−11.99	−8.28	−4.59	−0.92	2.72	6.35	9.95
		$\Delta_r G_m^o$	−0.66	−0.46	−0.23	0.02	0.27	0.53	0.79	1.05	1.31	1.57	1.83	2.08	2.34
100	9FO-S1 → 9FO-S2	$\Delta G^\ddagger$	31.98	32.52	33.07	33.66	34.26	34.88	35.50	36.14	36.78	37.43	38.09	38.75	39.42
		$\Delta_r G_m^o$	23.56	23.68	23.76	23.83	23.90	23.95	24.00	24.04	24.07	24.11	24.14	24.17	24.20
101	9FO-S2 → 9FO-S3	$\Delta G^\ddagger$	5.47	5.18	4.92	4.67	4.45	4.24	4.06	3.90	3.75	3.62	3.50	3.40	3.32
		$\Delta_r G_m^o$	−14.26	−18.24	−22.11	−25.94	−29.73	−33.49	−37.20	−40.89	−44.54	−48.17	−51.77	−55.34	−58.90
102	9FO-S3 → DF + ·OH	$\Delta G^\ddagger$	13.65	13.70	13.77	13.84	13.93	14.04	14.17	14.31	14.46	14.63	14.81	15.01	15.22
		$\Delta_r G_m^o$	−70.44	−72.63	−75.16	−77.65	−80.10	−82.52	−84.90	−87.26	−89.59	−91.89	−94.18	−96.44	−98.67
103	9FO-A2 → 9FO-S4	$\Delta G^\ddagger$	13.16	13.70	14.26	14.85	15.45	16.07	16.69	17.32	17.95	18.59	19.24	19.90	20.56
		$\Delta_r G_m^o$	−0.87	−0.83	−0.79	−0.74	−0.68	−0.62	−0.57	−0.51	−0.45	−0.40	−0.34	−0.29	−0.24
104	9FO-S4 → 9FO-S5 + CO_2	$\Delta G^\ddagger$	5.46	5.33	5.22	5.13	5.05	5.00	4.97	4.95	4.95	4.97	5.01	5.06	5.12
		$\Delta_r G_m^o$	−16.89	−20.67	−24.34	−27.97	−31.55	−35.10	−38.61	−42.09	−45.54	−48.97	−52.36	−55.73	−59.08
105	9FO-S5 + O_2 → Phe-S6	$\Delta_r G_m^o$	−33.37	−29.46	−25.64	−21.85	−18.10	−14.37	−10.68	−7.01	−3.36	0.26	3.85	7.43	10.98
106	9FO-S6 → 9FO-S3	$\Delta G^\ddagger$	29.78	30.29	30.79	31.30	31.81	32.32	32.83	33.35	33.87	34.40	34.93	35.47	36.02
		$\Delta_r G_m^o$	−4.31	−4.90	−5.52	−6.16	−6.82	−7.49	−8.18	−8.87	−9.57	−10.27	−10.98	−11.70	−12.42
107	9FO-S5 + O^{3P} → 9FO-S7	$\Delta_r G_m^o$	−115.49	−112.18	−108.90	−105.61	−102.33	−99.04	−95.76	−92.48	−89.20	−85.93	−82.67	−79.41	−76.16
108	9FO-S7 → 9FO-S8	$\Delta G^\ddagger$	28.23	28.76	29.33	29.94	30.58	31.24	31.93	32.64	33.37	34.11	34.87	35.64	36.43
		$\Delta_r G_m^o$	13.71	14.19	14.68	15.18	15.68	16.19	16.70	17.21	17.72	18.22	18.73	19.24	19.74

Table S8. *Cont.*

No.	Elementary Reactions		Temperature (K)												
			**298.15**	**400**	**500**	**600**	**700**	**800**	**900**	**1000**	**1100**	**1200**	**1300**	**1400**	**1500**
109	9FO-S8 → DF + ·H	$\Delta G^{\ddagger}$	21.24	21.34	21.44	21.53	21.62	21.71	21.80	21.90	22.00	22.11	22.23	22.35	22.48
		$\Delta_r G_m^o$	6.76	4.89	2.55	0.19	−2.20	−4.61	−7.03	−9.45	−11.89	−14.33	−16.76	−19.20	−21.65
110	9FO-D2 + O_2 → 9FO-S9	$\Delta_r G_m^o$	−33.72	−29.64	−25.65	−21.69	−17.76	−13.86	−9.99	−6.14	−2.32	1.47	5.24	8.99	12.73
111	9FO-S9 → 9FO-S10 + CO_2	$\Delta G^{\ddagger}$	37.16	37.74	38.33	38.95	39.60	40.25	40.93	41.63	42.34	43.06	43.80	44.56	45.32
		$\Delta_r G_m^o$	16.86	−21.07	−21.87	−22.72	−23.59	−24.49	−25.42	−26.37	−27.33	−28.32	−29.32	−30.34	−31.37
112	9FO-S10 → 9FO-S11	$\Delta G^{\ddagger}$	6.69	6.50	6.33	6.18	6.04	5.93	5.83	5.75	5.69	5.64	5.60	5.59	5.58
		$\Delta_r G_m^o$	−14.30	−17.59	−20.73	−23.79	−26.77	−29.69	−32.55	−35.35	−38.10	−40.81	−43.47	−46.09	−48.68
113	9FO-S11 → DF + ClO·	$\Delta G^{\ddagger}$	14.63	14.04	13.43	12.80	12.15	11.50	10.85	10.19	9.53	8.87	8.20	7.54	6.87
		$\Delta_r G_m^o$	−101.54	−103.86	−106.49	−109.06	−111.59	−114.10	−116.58	−119.04	−121.48	−123.92	−126.33	−128.74	−131.14
114	9FO-D2 → 9FO-S12	$\Delta G^{\ddagger}$	23.50	24.09	24.70	25.34	26.00	26.69	27.40	28.12	28.87	29.63	30.41	31.20	32.00
		$\Delta_r G_m^o$	−46.39	−46.31	−46.24	−46.17	−46.11	−46.04	−45.98	−45.91	−45.85	−45.79	−45.72	−45.66	−45.60
115	9FO-S12 →9 FO-S13 + CO_2	$\Delta G^{\ddagger}$	7.13	7.25	7.39	7.55	7.72	7.92	8.14	8.37	8.62	8.89	9.17	9.47	9.78
		$\Delta_r G_m^o$	−14.74	−18.44	−22.04	−25.58	−29.09	−32.56	−35.99	−39.38	−42.75	−46.09	−49.40	−52.68	−55.95
116	9FO-S13 + O_2 → Phe-S14	$\Delta_r G_m^o$	−34.43	−30.59	−26.83	−23.10	−19.40	−15.74	−12.10	−8.50	−4.91	−1.36	2.18	5.69	9.18
117	9FO-S14 → 9FO-S15	$\Delta G^{\ddagger}$	32.22	32.81	33.40	34.00	34.60	35.20	35.81	36.41	37.03	37.64	38.27	38.90	39.53
		$\Delta_r G_m^o$	26.06	25.95	25.80	25.64	25.46	25.27	25.08	24.87	24.67	24.47	24.26	24.05	23.84
118	9FO-S15 → 1CDF + ·OH	$\Delta G^{\ddagger}$	13.38	13.36	13.36	13.37	13.40	13.45	13.51	13.59	13.68	13.79	13.91	14.05	14.20
		$\Delta_r G_m^o$	−72.65	−75.34	−77.95	−80.51	−83.04	−85.54	−88.00	−90.44	−92.84	−95.23	−97.59	−99.92	−102.24
119	9FO-S13 + O^{3P} → 9FO-S16	$\Delta_r G_m^o$	−115.77	−112.46	−109.20	−105.92	−102.65	−99.38	−96.11	−92.84	−89.58	−86.33	−83.08	−79.83	−76.59
120	9FO-S16 → 9FO-S17	$\Delta G^{\ddagger}$	27.25	27.80	28.39	29.01	29.67	30.36	31.08	31.81	32.56	33.33	34.11	34.91	35.73
		$\Delta_r G_m^o$	11.44	11.90	12.38	12.88	13.37	13.88	14.38	14.88	15.39	15.89	16.40	16.90	17.40
121	9FO-S17 → 1CDF + ·H	$\Delta G^{\ddagger}$	21.36	21.47	21.56	21.65	21.74	21.83	21.92	22.01	22.12	22.22	22.34	22.46	22.59
		$\Delta_r G_m^o$	6.63	4.18	1.70	−0.81	−3.34	−5.90	−8.47	−11.05	−13.63	−16.22	−18.81	−21.40	−23.99
122	9FO-S16 → DF + ·Cl	$\Delta G^{\ddagger}$	20.59	20.66	20.74	20.85	20.98	21.13	21.29	21.48	21.69	21.91	22.14	22.40	22.66
		$\Delta_r G_m^o$	−30.48	−32.68	−35.23	−37.75	−40.25	−42.72	−45.18	−47.61	−50.04	−52.45	−54.85	−57.24	−59.61
